# CD8^+ ^lymphocyte infiltration is an independent favorable prognostic indicator in basal-like breast cancer

**DOI:** 10.1186/bcr3148

**Published:** 2012-03-15

**Authors:** Shuzhen Liu, Jonathan Lachapelle, Samuel Leung, Dongxia Gao, William D Foulkes, Torsten O Nielsen

**Affiliations:** 1 Genetic Pathology Evaluation Centre, Department of Pathology and Laboratory Medicine, University of British Columbia, 2660 Oak Street, Vancouver, BC, V6H 3Z6, Canada; 2 Department of Pathology, McGill University, 3775 University Street, Montreal, QC, H3A 2B2, Canada; 3 Program in Cancer Genetics, Department of Oncology and Human Genetics, McGill University, 546 Pine Avenue West, Montreal, QC, H2W 1S6, Canada; 4 Department of Medical Genetics, Lady Davis Institute, Segal Cancer Centre, Jewish General Hospital, 3755 Chemin de la Côte-Sainte-Catherine, Montreal, QC, H3T 1E2, Canada; 5 Medical Genetics and Genomics Axis, Research Institute of the McGill University Health Centre, 1650 Cedar Avenue, Montreal, QC, H3G 1A4, Canada

## Abstract

**Introduction:**

Tumor infiltrating lymphocytes may indicate an immune response to cancer development, but their significance remains controversial in breast cancer. We conducted this study to assess CD8+ (cytotoxic T) lymphocyte infiltration in a large cohort of invasive early stage breast cancers, and to evaluate its prognostic effect in different breast cancer intrinsic subtypes.

**Methods:**

Immunohistochemistry for CD8 staining was performed on tissue microarrays from 3992 breast cancer patients. CD8+ tumor infiltrating lymphocytes were counted as intratumoral when in direct contact with tumor cells, and as stromal in adjacent locations. Kaplan-Meier functions and Cox proportional hazards regression models were applied to examine the associations between tumor infiltrating lymphocytes and breast cancer specific survival.

**Results:**

Among 3403 cases for which immunohistochemical results were obtained, CD8+ tumor infiltrating lymphocytes were identified in an intratumoral pattern in 32% and stromal pattern in 61% of the cases. In the whole cohort, the presence of intratumoral tumor-infiltrating lymphocytes was significantly correlated with young age, high grade, estrogen receptor negativity, human epidermal growth factor receptor-2 positivity and core basal intrinsic subtype, and was associated with superior breast cancer specific survival. Multivariate analysis indicated that the favorable prognostic effect of CD8+ tumor infiltrating lymphocytes was significant only in the core basal intrinsic subgroup (Hazard ratio, HR = 0.35, 95% CI = 0.23-0.54). No association with improved survival was present in those triple negative breast cancers that lack expression of basal markers (HR = 0.99, 95% CI = 0.48-2.04) nor in the other intrinsic subtypes.

**Conclusions:**

CD8+ tumor infiltrating lymphocytes are an independent prognostic factor associated with better patient survival in basal-like breast cancer, but not in non-basal triple negative breast cancers nor in other intrinsic molecular subtypes.

## Introduction

Immune response may play an important role in cancer progression. Tumor-infiltrating lymphocytes (TILs) reflect a local immune response and could be a key mechanism in controlling tumor progression [1,2]. A number of studies demonstrate that TILs are associated with clinical outcome in patients with carcinoma and melanoma [3-8]. TILs have been found to be mainly T lymphocytes, and the majority express a cytotoxic effector phenotype (CD8^+^) [9-11]. CD8^+ ^T cell-mediated type 1 immune responses can enhance the accumulation of distinct endogenous CD8^+ ^and CD4^+ ^T cells and facilitate their antitumor function within the tumor microenvironment [12,13]. Studies in ovarian carcinomas and colon cancer show that high levels of CD8^+ ^lymphocyte infiltration are associated with better prognosis in these diseases [3,14]. In breast cancer, some studies have reported that inflammation and cytotoxic lymphocyte infiltration are associated with better survival [15-17]. In contrast, other groups have reported that high numbers of TILs are related to worse overall survival [18,19], whereas still other studies did not find any significant association of TILs with patient outcome [20,21]. A recent publication reported that a high ratio of CD8^+ ^TILs to FOXP3^+ ^regulatory T cells had a significant relationship to improved patient survival in breast cancer [22]. Two other studies have tested larger series: one study used a retrospective cohort of 1,334 patients with primary breast cancer diagnosed from 1987 to 1998 in the UK and showed that total CD8^+ ^TILs were independently associated with better survival in breast cancer [23], whereas another study with 1,953 breast cancer cases treated in the University Hospital Basel in Switzerland between 1985 and 1996 demonstrated that the independent favorable prognostic effect of total CD8^+ ^TILs was observed only in those with estrogen receptor-negative (ER^-^) tumors (whereas, in univariate analyses, CD8^+ ^TILs had an unfavorable effect on outcome in ER-positive (ER^+^) breast cancers) [24]. Thus, the extent to which TILs contribute to tumor progression and clinical outcome in breast cancer has remained controversial, possibly because the effect is limited to certain subgroups of patients.

Breast cancer is a heterogeneous disease composed of different intrinsic subtypes, each with distinctive biological and prognostic behaviors and responses to therapy. Although the introduction of adjuvant systemic therapy (AST) has led to a significant reduction in breast cancer mortality, many patients do not benefit. Gene expression studies suggest that predictive indicators should be developed for different breast cancer subtypes [25,26]. The interaction between immune response, intrinsic subtype, and treatment strategy all likely contribute to the outcome of the disease. The development of molecular diagnostic techniques has facilitated a better understanding of the heterogeneity of breast cancer and opened up the possibility of more personalized therapy [27,28]. Hormone receptor status and human epidermal growth factor receptor-2 (HER2) molecular status are currently used to guide AST strategies for the luminal and HER2^+ ^intrinsic subgroups, but no targeted therapy for the basal-like subgroup is currently available. Basal-like breast cancer comprises about 15% of all invasive breast cancers and is likely to be high-grade, occur in young women, and have an aggressive clinical course [29]. Although a majority of basal-like tumors carry a clinical triple-negative phenotype (TNP) (ER^-^, progesterone receptor-negative (PR^-^), and HER2^-^), they are not synonymous [30], and triple-negative breast cancers include many cases that lack the expression of basal markers - the so-called 'five-marker negative phenotype' (5NP): ER^-^, PR^-^, HER2^-^, epidermal growth factor receptor-negative (EGFR^-^), and cytokeratin (CK) 5/6^- ^- which have been shown to have significantly better outcomes than core basal cases [31,32]. Gene expression profiling data suggest that medullary breast tumors (a rare histological subtype with a prominent lymphocytic reaction and a good prognosis) are a specific subgroup within the basal-like class, indicating that the overall poor survival of basal-like breast cancer might be mitigated in cases in which there is a strong immune response [33-35]. On the other hand, a separate body of research has highlighted that recruitment of chronic inflammatory cells, including macrophages, can actually promote cancer progression [36]. Different types of immune response in different subtypes of breast cancer might explain apparently contradictory results. However, to date, no large immunohistochemistry study has explored the prognostic effect of an immune response in breast cancer stratified by breast cancer intrinsic subtype.

Therefore, there is a clear need for studies with sufficient power for subgroup analysis, employing validated measurements of immune response, to evaluate the significance of TILs in breast tumors. The aim of this study was to examine the prognostic significance of CD8^+ ^TILs in different breast cancer intrinsic subtypes in a large population-based cohort with long-term follow-up. Our hypothesis was that CD8^+ ^lymphocyte infiltration has distinct prognostic effects in different intrinsic molecular subtypes of breast cancer.

## Materials and methods

### Study population

The study population consists of 3,992 female patients with invasive breast cancer diagnosed between 1986 and 1992 in the province of British Columbia. This cohort was collected from the Breast Cancer Outcomes Unit database maintained by the British Columbia Cancer Agency (BCCA). During the study period, 75% of patients with breast cancer in the province were referred to the BCCA; non-referred patients were generally older or had no indications for adjuvant therapy [37,38]. Of the patients referred to the BCCA, approximately 25% had available formalin-fixed paraffin-embedded blocks with sufficient tumor tissues for tissue microarray (TMA) construction. Thus, the study cohort represents about 20% of all of the patients with breast cancer diagnosed in the province during the study period. The mean age of the cohort at diagnosis was 58.9 years (23 to 95 years), and the median follow-up was 12.6 years. Baseline clinical information of the study population includes age at diagnosis, histology, grade, tumor size, number of involved axillary nodes, lymphovascular invasion (LVI), and dates of diagnosis, recurrence, death, and cause of death (breast cancer versus other). As shown in Table 1 among the study cases, approximately half (51.1%, 2,040/3,992) were poorly differentiated tumors (grade 3), 47.3% (1,888/3,992) had breast tumors over 2 cm, 43.1% (1,719/3,992) were node-positive, and 42.8% (1,710/3,992) had LVI. Histological categorization on these cases, including assignment to the medullary subtype, was determined by a central review of full sections which was performed at the time of referral to the BCCA. During the time period of this study cohort, most patients with breast cancer were treated according to the provincial guidelines developed by the BCCA on the basis of patient age, tumor size, nodal status, and LVI. Patients were defined as high-risk if their lymph nodes were positive, if there was evidence of LVI, or if the tumor was both greater than 2 cm and ER^- ^at the time of diagnosis. High-risk patients were treated with AST according to their age and menopausal status. Low-risk patients were not given any AST. This study and the use of de-identified data were approved by the Clinical Research Ethics Board of the BCCA and the University of British Columbia. We were permitted access to the de-identified patient outcome information from the Breast Cancer Outcomes Unit database, maintained by the BCCA. In compliance with the Canadian Tri-Council Policy Statement for ethical research involving human subjects, the requirement for informed consent was waived as this study was limited to anonymous archival specimens.

**Table 1:** Clinicopathologic characteristics and distribution of CD8^+ ^intratumoral lymphocytes in the study population

Characteristics	Patients, number (percentage)	iTILs (≥ 1)	
		Prevalence, percentage (proportion)	*P *value
Age at diagnosis, years			< 0.001
< 40	294 (7.4)	38.9 (98/252)	
40-49	844 (21.1)	37.5 (273/728)	
50-65	1,425 (35.7)	31.3 (377/1,203)	
> 65	1,429 (35.8)	29.2 (356/1,220)	
Grade			< 0.001
1: well differentiated	209 (5.2)	24.4 (40/164)	
2: moderately well or partially differentiated	1,563 (39.2)	26.9 (361/1,342)	
3: poorly differentiated	2,040 (51.1)	37.2 (652/1,754)	
Unknown	180 (4.5)		
Tumor size, centimeters			0.076
≤ 2	2,078 (52.1)	30.5 (540/1,768)	
> 2-5	1,667 (41.8)	34.1 (494/1,449)	
> 5	221 (5.5)	34.9 (59/169)	
Unknown	26 (0.6)		
Nodal status			0.051
Negative	2,265 (56.7)	31.0 (593/1,911)	
Positive	1,719 (43.1)	34.3 (509/1,484)	
Unknown	8 (0.2)		
Lymphovascular invasion			0.638
Negative	2,106 (52.8)	32.5 (576/1,770)	
Positive	1,710 (42.8)	31.8 (474/1,492)	
Unknown	176 (4.4)		
Histology			< 0.001
Medullary	66 (1.7)	78.4 (40/51)	
Not medullary	3926 (98.3)	31.7 (1,064/3,352)	
AJCC stage			0.004
I	1,393 (34.9)	28.8 (337/1,172)	
II	2,255 (56.5)	34.6 (677/1,959)	
III	317 (7.9)	32.9 (83/252)	
Unknown/missing	27 (0.7)		
Adjuvant systemic therapy			0.012
No adjuvant systemic therapy	1,676 (42.0)	21.2 (302/1,427)	
Tamoxifen only	1,276 (32.0)	18.6 (206/1,105)	
Chemotherapy only	727 (18.2)	27.2 (169/622)	
Tamoxifen + chemotherapy	297 (7.4)	29.4 (73/148)	
Other	16 (0.4)	21.4 (3/14)	
ER			< 0.001
Negative	1,200 (30.1)	39.9 (370/927)	
Positive (≥ 1% nuclei stained)	2,761 (69.1)	29.6 (728/2,456)	
Uninterpretable/missing	31 (0.8)		
HER2			< 0.001
Negative	3,316 (83.1)	31.3 (907/2,902)	
Positive	498 (12.5)	39.6 (176/444)	
Uninterpretable/missing	178 (4.4)		
Subtype			< 0.001
Luminal A	1,518 (38.0)	25.4 (353/1,392)	
Luminal B	829 (20.8)	36.9 (285/773)	
Luminal/HER2	224 (5.6)	39.8 (82/206)	
Luminal not further assigned	244 (6.1)	21.5 (37/172)	
HER2^+^/ER^-^	250 (6.3)	39.6 (90/227)	
TNP	630 (15.8)	42.2 (226/535)	
Core basal	330 (8.3)	49.2 (151/307)	
5NP	162 (4.1)	35.2 (50/142)	
TNP not assignable	138 (3.4)	29.1 (25/86)	
Unassignable	297 (7.4)	31.6 (31/98)	
Total	3,992 (100)	32.4 (1,104/3,403)	

5NP, five negative phenotype; AJCC, American Joint Committee on Cancer; ER, estrogen receptor; HER2, human epidermal growth factor receptor-2; iTIL, intratumoral tumor-infiltrating lymphocyte; TNP, triple-negative phenotype.

### Tissue microarray and immunohistochemistry

The centralized provincial laboratory of Vancouver General Hospital retained single archival blocks for each of the 3,992 patients. One 0.6-mm core per patient was used, 17 TMAs representing these samples were constructed, and immunohistochemistry and scoring for ER, PR, HER2, the Ki67 proliferation marker, EGFR, and CK5/6 were performed as previously described [31,37,39-42]. Immunohistochemistry for CD8^+ ^TILs was performed by using the antibody against human CD8 (clone C8/144B, dilution 1:100) in accordance with the protocol of the manufacturer (DakoCytomation, Glostrup, Denmark). Intrinsic breast cancer subtypes were determined by the immunohistochemical expressions of ER, PR, HER2, Ki67, EGFR, and CK5/6. Luminal A was defined as ER^+ ^or PR^+^, HER2^-^, and low Ki67 (< 14%); luminal B was defined as ER^+ ^(or PR^+^) and HER2^- ^with high Ki67 (≥ 14%); luminal/HER2 subgroup was defined as ER^+ ^(or PR^+^) and HER2^+^; HER2^+^/ER^- ^was defined as HER2^+ ^with ER^- ^and PR^- ^[42]; and triple-negative subgroup (TNP) was defined as ER^-^, PR^-^, and HER2^-^. The core basal subgroup was defined as triple-negative with either EGFR^+ ^or CK5/6^+^, and the five negative phenotype (5NP) was defined as triple-negative as well as EGFR^- ^and CK5/6^- ^[31]. The 3,992 patients with breast cancer were thereby categorized as follows: 38.0% (1,518/3,992) luminal A, 20.8% (829/3,992) luminal B, 5.6% (223/3,992) luminal/HER2, 6.3% (250/3,992) HER2^+^/ER^-^, and 15.8% (630/3,992) triple-negative, of which 8.3% (330/3,992) could be categorized as core basal and 4.1% (162/3,992) as 5NP; the remainder had a partial or unassignable subtype because of missing or ambiguous biomarker data (Table 1).

### CD8^+ ^tumor-infiltrating lymphocytes: scoring and quantification

Stained TMA slides were digitally scanned and CD8^+ ^TILs were visually scored by a pathologist who was blinded to the clinical characteristics and outcomes of the patients. Scoring and quantification of CD8^+ ^TILs were carried out as described in a recent study [24]. In brief, intratumoral CD8^+ ^TILs (iTILs) were defined as CD8^+ ^lymphocytes located within tumor cell nests or in direct contact with the breast carcinoma malignant epithelial cells, whereas stromal CD8^+ ^TILs (sTILs) were defined as CD8^+ ^lymphocytes in the adjacent peritumoral stroma without direct contact with the carcinoma cells. Total CD8^+ ^TILs (tTILs) were measured by combining the counts of iTILs and sTILs for each tissue core. To assess the reproducibility and reliability of the scoring, 490 cases were repeatedly scored by the same pathologist after a period of time (4 weeks), and 200 cases were randomly selected from the whole cohort and iTILs were re-scored by a second pathologist. Pearson correlation analysis was used to check the reliability of the repeated scoring by the same scorer, and the intraclass correlation coefficient (ICC) was used to assess the reliability of re-scoring by the two scorers. High correlation coefficients were obtained (Pearson r was at least 0.94, and ICC was 0.74).

### Statistical analysis

The outcome variable in this study was breast cancer-specific survival (BCSS). Optimal cutoff points for TILs counts against BCSS were chosen on the basis of recently published findings from an independent series [24] and checked by receiver operating characteristic curve analysis by using 10-year BCSS as the endpoints, as described in the Supplemental method section (Additional file 1). The optimal cutoff points for iTIL, sTIL, and tTIL used in this study were 1, 3, and 2, respectively. To specify, CD8^+ ^iTIL expression was categorized as low when iTIL was 0 (no CD8^+ ^iTIL counted) and high when iTIL was at least 1 (1 or more CD8^+ ^iTILs in the assessed tissue core); sTIL low means fewer than 3 CD8^+ ^sTILs per core, and tTIL low means fewer than 2 CD8^+ ^tTILs were identified in a core.

Analysis of the association between TILs and clinicopathologic variables was performed by using SPSS version 19.0 and R 2.11.1. Because the distributions of the outcome variable (BCSS) were not normal in the study cohort, non-parametric Wilcoxon testing was used to check the bivariate relationship between BCSS and TILs and other potential confounding variables, including age at diagnosis, grade, tumor size, involvement of lymph nodes, LVI, and intrinsic subtypes. Chi-squared testing was used to check the relationship between TILs and those potential confounding variables. For survival analysis, the event under study was death from breast cancer. BCSS time was defined as the number of years between the date of diagnosis of breast cancer and the date of death attributable to breast cancer. Survival time was censored at the time a patient died from another cause or when the follow-up period ended. For univariate survival analyses, the Kaplan-Meier function analysis was performed to estimate probabilities of BCSS. Log-rank testing was used to assess differences in BCSS among different subgroups. For multivariate survival analyses, Cox proportional hazards regression models were built to estimate the TIL hazard ratio (HR), which was adjusted by the potential confounding variables on the basis of the partial maximum likelihood estimation. Smoothed, rescaled Schoenfeld residual plots were performed to test proportional hazards assumptions. Only cases with sufficient information for all covariates were included in the multivariate analysis. Wald statistics were used to test the significance of individual coefficients. Interactions between TILs and some covariables were checked by building Cox regression models for different levels of those variables and comparing HRs of TILs. All of the tests were two-sided at a significance level of 0.05. Supplementary analyses were also performed by using relapse-free survival as an outcome variable; relapse-free survival time was defined as the number of years between the date of diagnosis of breast cancer and the date of any type of relapse, including local, regional, and distant relapses of the disease.

## Results

### CD8^+ ^tumor-infiltrating lymphocyte counts and distributions in breast cancer

Among the 3,992 breast tumor cases, intact cores bearing infiltrating breast carcinoma sufficient for interpretation of immunohistochemical data for CD8 staining were available from 3,403 (85.2%) tumors. Median counts of CD8^+ ^TILs per 0.6-mm TMA core were 0 for iTIL (interquartile range, or IQR, of 0 to 1), 2 for sTIL (IQR of 0 to 10), and 3 for tTIL (IQR of 0 to 12). Of the 3,403 interpretable cases, 32.4% had tumor infiltrated with at least one CD8^+ ^iTIL and 60.6% by at least one CD8^+ ^sTIL (Figure S1 of Additional file 2). The distributions of CD8^+ ^iTILs and sTILs were both significantly and positively skewed (Figure S2 of Additional file 3). Because analytical results from all types of TILs interpretation were broadly similar, results presented in this paper are based primarily on iTILs analysis, which is the fastest and simplest to perform. As shown in Table 1 the presence of iTIL is significantly associated with young age, high grade, medullary histology, ER negativity, HER2 positivity, and the core basal intrinsic subgroup, the category that has the highest prevalence of cases displaying intratumoral lymphocytes.

### Prognosis of CD8^+ ^iTILs in patients with breast cancer (whole cohort)

**Figure 1:**
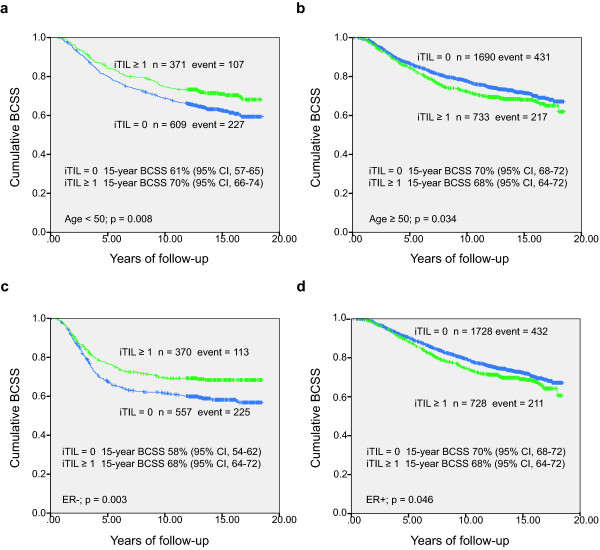
**Breast cancer-specific survival (BCSS) by intratumoral tumor-infiltrating lymphocytes (iTILs) among groups with different age and estrogen receptor (ER) status. (a) **Age of less than 50 years, **(b) **age of at least 50 years, **(c) **ER^-^, and **(d) **ER^+^. CI, confidence interval.

To examine the prognosis of CD8^+ ^TILs in the study population, we first applied univariate Kaplan-Meier function survival analysis in the whole cohort. The results did not show a significant difference in BCSS between breast cancer patients with an iTIL count of at least 1 and an iTIL of 0 (*P *= 0.761). Since the distribution of iTILs was associated with patient age at diagnosis, tumor grade, and ER and HER2 status, we next assessed the survival functions of iTIL associated with BCSS in groups with different age, tumor grade, and ER and HER2 status. Figure 1 showed that, in younger patients (< 50 years) and in those with ER^- ^tumors, cases with iTILs had significantly better BCSS than those without. Reversed associations were observed in patients who were at least 50 years old or who had ER^+ ^breast cancer. No significant associations were detected in cases stratified by grade (grade 1 + 2 versus grade 3) or HER2 status (HER2^+ ^versus HER2^-^). These results indicated that age and ER status could have an interaction with the association between iTILs and patient survival in breast cancer.

We built Cox proportional hazards regression models to estimate the HR for iTILs. Smoothed, rescaled Schoenfeld residual plots showed that iTILs and most other covariables satisfied the proportional hazards assumptions well during the period of follow-up. Only iTILs in the luminal A subgroup varied slightly during longer follow-up.

Results from the univariate Cox regression model analysis showed that iTILs was not a significant prognostic factor associated with BCSS in the cohort as a whole: HR = 1.02, 95% confidence interval (CI) = 0.89 to 1.17. To take into consideration potential confounders, a multivariate Cox regression model was built to assess the association between iTILs and BCSS, including the covariates of age at diagnosis, tumor grade and size, lymph node status, LVI, and intrinsic subtype. Table 2 showed that the adjusted HR of iTIL was 0.79 (95% CI = 0.68 to 0.91), meaning that, in the whole cohort, the probability of BCSS among patients with an iTIL count of at least 1 was 21% (1 to 0.79) higher than among those with an iTIL count of 0 after adjustment for age, grade, tumor size, lymph node status, LVI, and intrinsic subtypes. Besides iTIL, tumor grade and size, nodal status, LVI, and intrinsic subtype, each had significant effects on BCSS. To examine the effect of interaction between age, ER status, and iTIL, we built multivariate Cox regression models for iTILs at different levels of age and ER status. These analyses showed that the adjusted HRs for iTILs were 0.65 (95% CI = 0.51 to 0.84) for those younger than 50 years old and 0.89 (95% CI = 0.74 to 1.06) for those at least 50 years old; the adjusted HRs were 0.61 (95% CI = 0.47 to 0.77) for those with ER^- ^tumors but 0.91 (95% CI = 0.77 to 1.11) for those with ER^+ ^tumors. Therefore, interactions between iTIL and age and ER status might modify the effect size for iTILs in the unstratified whole cohort of patients with breast cancer.

**Table 2:** Hazards for breast cancer-specific survival in the whole cohort with univariate and multivariate analyses

Variable	Univariate analysis		Multivariate analysis *n *= 3,144	
	HR (95% CI)	*P *value	HR (95% CI)	*P *value
Age				
≥ 50 vs. < 50 years	0.85 (0.75-0.96)	0.011	1.01 (0.88-1.16)	0.884
Grade				
3 vs. 1 and 2	2.12 (1.87-2.41)	< 0.001	1.57 (1.35-1.82)	< 0.001
Tumor size				
> 2 vs. ≤ 2 cm	2.17 (1.92-2.45)	< 0.001	1.59 (1.36-1.83)	< 0.001
Nodal status				
Positive vs. negative	2.79 (2.48-3.15)	< 0.001	2.05 (1.76-2.39)	< 0.001
Lymphovascular invasion				
Positive vs. negative	2.25 (1.99-2.54)	< 0.001	1.29 (1.10-1.51)	0.001
Subtype				
Luminal B vs. luminal A	2.08 (1.78-2.45)	< 0.001	1.75 (1.46-2.09)	< 0.001
HER2^+^/ER^- ^vs. luminal A	2.98 (2.40-3.70)	< 0.001	2.51 (2.99-3.19)	< 0.001
Core basal vs. luminal A	2.30 (1.87-2.84)	< 0.001	2.02 (1.58-2.58)	< 0.001
5NP vs. luminal A	1.65 (1.30-2.10)	0.002	1.49 (1.12-1.97)	0.011
iTIL				
≥ 1 vs. 0	1.02 (0.89-1.17)	0.761	0.79 (0.68-0.91)	< 0.001

5NP, five negative phenotype; CI, confidence interval; ER, estrogen receptor; HER2, human epidermal growth factor receptor-2; HR, hazard ratio; iTIL, intratumoral tumor-infiltrating lymphocyte.

### Association of CD8^+ ^iTILs with breast cancer-specific survival in different breast cancer intrinsic subgroups

**Figure 2:**
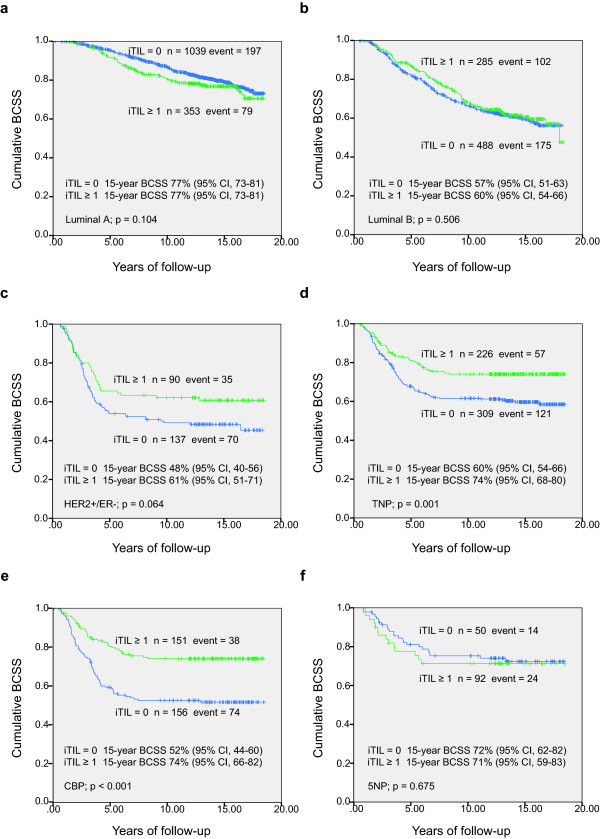
**Breast cancer-specific survival (BCSS) by intratumoral tumor-infiltrating lymphocytes (iTILs) in different breast cancer intrinsic subgroups. (a) **Luminal A, **(b) **luminal B, **(c) **HER2^+^/ER^-^, **(d) **triple-negative phenotype, **(e) **core basal phenotype, and **(f) **five negative phenotype subgroups. CI, confidence interval; ER, estrogen receptor; HER2, human epidermal growth factor receptor-2.

We further assessed the association of CD8^+ ^TILs with patient survival in different breast cancer intrinsic subtypes, first using univariate Kaplan-Meier function survival analysis. No difference in BCSS was detected between those with an iTIL count of at least 1 and an iTIL count of 0 within the luminal A and luminal B subgroups (Figure 2a, b). Although we observed an apparent difference between the two groups among HER2^+^/ER^- ^cases, this was not statistically significant (*P *= 0.064) (Figure 2c). However, as shown in Figure 2d, a large and significant difference in BCSS was found between cases with an iTIL count of at least 1 and those with an iTIL count of 0 among triple-negative breast cancers. By stratifying triple-negatives into core basal and 5NP subgroups, we observed a much larger difference in BCSS between cases with an iTIL count of at least 1 and those with an iTIL count of 0 in the core basal intrinsic subgroup. Patients with an iTIL count of at least 1 basal-like tumor had significantly better survival than those with an iTIL count of 0 (mean survival time of 14.5 vears versus 11.0 years, *P *< 0.001) (Figure 2e). No such association was found among triple-negative, non-basal (5NP) cases (Figure 2f). We also performed survival analysis in all patients with ER^- ^breast cancer, excluding the core basal cases, and found no significant difference in BCSS between cases with an iTIL count of at least 1 and those with an iTIL count of 0 (*P *= 0.434).

To confirm the association between iTIL and BCSS and to assess the independent prognostic effect size in different breast cancer intrinsic subgroups, multivariate Cox proportional hazards regression models were built to estimate the iTIL HRs, which were adjusted by the potential confounders. Results in Table 3 showed that the HRs of iTIL were not significant in the luminal A, luminal B, and HER2^+^/ER^- ^intrinsic subgroups. However, iTIL was demonstrated to be a significantly independent favorable factor for BCSS in triple-negative cases because of a strong effect in the core basal subgroup (Table 4). Among core basal cases, the presence of any intratumoral CD8^+ ^lymphocytes (iTILs of at least 1) was associated with a 65% higher probability of BCSS than among those tumors lacking intratumoral CD8^+ ^lymphocytes (iTIL of 0) and this was statistically significant even after adjusting for age at diagnosis, grade, tumor size, lymph node status, and LVI. Considering that medullary breast carcinoma, a histologically evident subtype known to carry a good prognosis, usually has a core basal immunophenotype and could be responsible for some of the observed effect, we repeated the multivariate Cox regression analysis for core basal cases by excluding those with medullary carcinoma (27 cases). The results still showed a similar and significant HR (HR = 0.38, 95% CI = 0.24 to 0.59), which therefore could not be attributed to medullary histology. In contrast, the multivariate analysis did not show any association between iTILs and BCSS in the 5NP subgroup (that is, triple-negative breast cancers that do not express basal markers). These results demonstrated that the prognostic effect of iTILs was significantly different in these two subgroups of triple-negative cases, indicating that the association of iTIL with BCSS exists primarily in only the core basal intrinsic subgroup.

**Table 3:** Hazards for breast cancer-specific survival with multivariate analysis in the luminal A, luminal B, and HER2^+^/ER^- ^intrinsic subgroups

Variable	Luminal A (*n *= 1,276)		Luminal B (*n *= 709)		HER2^+^/ER^- ^(*n *= 216)	
	HR	*P*	HR	*P*	HR	*P*
Age	1.38		1.04		1.13	
≥ 50 vs. < 50 years	(1.02-1.86)	0.037	(0.81-1.35)	0.750	(0.75-1.70)	0.564
Grade	1.75		1.28		2.13	
3 vs. 1 and 2	(1.36-2.25)	< 0.001	(0.99-1.67)	0.062	(1.21-3.76)	0.009
Tumor size	1.64		1.49		1.73	
> 2 vs. ≤ 2 cm	(1.28-2.11)	< 0.001	(1.14-1.95)	0.004	(1.11-2.68)	0.015
Nodal status	2.20		1.75		1.75	
Positive vs. negative	(1.65-2.95)	< 0.001	(1.31-2.32)	< 0.001	(1.07-2.83)	0.025
Lymphovascular invasion	1.12		1.33		1.36	
Positive vs. negative	(0.84-1.49)	0.444	(0.99-1.77)	0.056	(0.84-2.18)	0.211
iTIL	1.14		0.85		0.76	
≥ 1 vs. 0	(0.86-1.50)	0.357	(0.66-1.11)	0.235	(0.50-1.15)	0.194

ER, estrogen receptor; HER2, human epidermal growth factor receptor-2; HR, hazard ratio; iTIL, intratumoral tumor-infiltrating lymphocyte.

**Table 4:** Hazards for breast cancer-specific survival with multivariate analysis in TNP, core basal, and 5NP groups

Variable	TNP (*n *= 496)		Core basal (*n *= 287)		5NP (*n *= 130)	
	HR	*P*	HR	*P*	HR	*P*
Age	0.90		0.91		1.07	
≥ 50 vs. < 50 years	(0.66-1.22)	0.488	(0.62-1.35)	0.648	(0.54-2.14)	0.830
Grade	1.74		1.54		1.81	
3 vs. 1 and 2	(1.11-2.70)	0.015	(0.80-2.97)	0.201	(0.74-4.41)	0.191
Tumor size	1.66		1.85		1.49	
> 2 vs. ≤ 2 cm	(1.19-2.30)	0.003	(1.23-2.79)	0.003	(0.71-3.12)	0.293
Nodal status	2.00		2.16		1.58	
Positive vs. negative	(1.42-2.83)	< 0.001	(1.39-3.35)	0.001	(0.73-3.42)	0.244
Lymphovascular invasion	1.55		1.52		3.13	
Positive vs. negative	(1.08-2.21)	0.017	(0.97-2.36)	0.065	(1.27-7.77)	0.013
iTIL	0.48		0.35		0.99	
≥ 1 vs. 0	(0.34-0.67)	< 0.001	(0.23-0.54)	< 0.001	(0.48-2.04)	0.986

5NP, five negative phenotype; HR, hazard ratio; iTIL, intratumoral tumor-infiltrating lymphocyte; TNP, triple-negative phenotype.

### Association of CD8^+ ^sTILs and tTILs with clinical outcome

To confirm the prognostic value of CD8^+ ^TILs in breast cancer, we also evaluated the distributions of sTILs and tTILs in relation to patient and tumor characteristics and the associations of sTILs and tTILs with survival. Results similar to those from the analysis with iTILs were obtained. In brief, high expressions of sTILs and tTILs were significantly correlated with young age, high grade, larger tumor size, medullary histology, ER negativity, HER2 positivity, and the core basal phenotype (Table S1 of Additional file 4) and again were significantly associated with better BCSS in only the core basal intrinsic subgroup (Figure S3 of Additional file 5 and Tables S2 and S3 of Additional file 4).

## Discussion

The prognostic significance of TILs in breast cancer has been debated, but no consistent conclusion has yet been drawn. We implemented this study, using a particularly large, well-annotated cohort comprising nearly 4,000 patients, in an attempt to definitively assess the clinical implication of TILs in breast cancer. In addition to addressing the question of whether immune response (as measured by CD8^+ ^TILs) has a prognostic role in breast cancer in general, we examined the effect of TILs in the major breast cancer intrinsic biological subtypes. To our knowledge, this is the first study sufficiently powered for multivariate analysis to investigate the association of CD8^+ ^TILs with patient survival within the breast cancer intrinsic subtypes. Our results demonstrate that the presence of iTILs is independently associated with a significantly superior outcome in women with diagnosed core basal tumors. Although the presence of CD8^+ ^iTILs is also an independent prognostic indicator for improved patient survival in triple-negative breast cancers, this favorable prognostic effect cannot be detected among those lacking expression of basal biomarkers (5NP). In the core basal subgroup, patients having tumors with CD8^+ ^iTILs survived an average of 3.5 years longer than did patients with basal tumors lacking evidence of a CD8^+ ^iTIL immune response.

Breast cancer is both clinically and molecularly heterogeneous and is, in practice, stratified by hormonal receptors (ER and PR), by HER2 status, and, increasingly, by expression of other biomarkers such as Ki67 or by gene expression profiling methodologies. Dissecting the heterogeneity of breast cancer is critically important for understanding the underlying mechanisms of the disease and for identifying subpopulations that are most likely to respond to particular therapies [43]. In general, ER^- ^breast cancers have a worse prognosis than those that are ER^+^, but not all patients with ER^- ^breast cancer have poor survival. Teschendorf and colleagues [44] applied an integrative analysis of three gene expression datasets to assess the prognostic value of molecular signatures and found that most prognostic markers of better prognosis in ER^- ^breast cancer are associated with the activation of immune response pathways. Furthermore, a seven-gene immune response classifier was constructed and showed significant good prognostic value in patients with ER^- ^breast cancer [45]. Meta-analytic studies of clinical and gene expression data have demonstrated that immune response is significantly associated with prognosis in breast cancer [46], primarily in rapidly proliferating [47] and ER^- ^[48,49] subgroups. Results from some studies indicate that TILs could be a protective factor reducing the likelihood of distant metastasis in patients with triple-negative breast tumors [50] and among those with medullary carcinoma [17]. Moreover, two recently published gene expression profiling studies demonstrated that effective immune (particularly cytotoxic T-cell) response plays a favorable prognostic role in basal breast cancer subgroups [51,52]. In our study, the multivariate analysis clearly demonstrates that the presence of CD8^+ ^iTILs has a different prognostic value in breast cancer with different intrinsic biological subtypes. Even among the triple-negative cases, immune response has different meanings in core basal versus 'five negative' phenotypes. Evidence from previous studies has shown that core basal-like tumors are associated with a poorer prognosis and appear biologically different from 5NP tumors [31,32]. Our results suggest that local immune response characterized by CD8^+ ^lymphocyte infiltration might be considered an important factor differentiating the core basal from 5NP breast tumors within the class of triple-negative breast cancers.

Tumor-infiltrating lymphocytes and macrophages are thought to be molecular determinants of clinical outcome in breast cancer. Although cytotoxic T lymphocytes and natural killer cells have been found to have antitumor activity, some lymphocytes such as B cells exhibit bipolar roles in breast cancer development. Distinct cell-mediated immune responses also play antagonistic roles in disease prognosis. T helper cell 1 (Th1)-mediated immune response pathways are considered to have an inhibitory effect, whereas Th2 immune response pathways may promote development and metastasis of breast cancer. It has been found that CD4^+ ^T lymphocytes can promote metastasis by activating the EGFR signaling pathway in a Th2-type tumor microenvironment [53]. Identification of interactions between immune response and other molecular pathways may define novel prognostic subtypes. In ER^- ^breast cancer, those characterized with high expression of EGFR and low expression of Th1-mediated pathway-related markers such as interleukin-12 and interferon-gamma were found to have a poor prognosis [54]. TILs in the tumor microenvironment are predominantly CD8^+ ^T cells [55,56], which are considered to be the effector cells in Th1 antitumor immune responses. CD8^+ ^T cells produce interferon-gamma through interaction with tumor-related antigens, potential leading to tumoricidal activity by induction of apoptosis or macrophage tumor killing activity or both [57]. Studies indicate that tumor-specific or even non-cancer-specific antigens such as p53 and β-actin are common targets of cytotoxic T lymphocytes and can induce immunological and clinical effects in patients with breast cancer [58-60]. Findings from our study suggest that core basal-like breast cancer is more immunogenic than other intrinsic subgroups, as measured by CD8^+ ^T-cell infiltration. Tumors of this subtype have a high expression of basal markers, some of which (such as EGFR) may interact with T cell-mediated immune response to affect clinical outcome in breast cancer. We would suggest a hypothesis that certain 'basal proteins' expressed on the cell surface can be recognized as tumor antigens and that the consequent induction of adaptive basal marker-specific immunity can enhance the local Th1-mediated antitumor immune response in these breast cancers. The absence of these surface markers in 5NP breast cancers could underpin the observed difference in prognostic significance of TILs in core basal compared with 5NP breast cancers.

Recent studies have suggested that a pre-existing immune response can strengthen the effect of conventional chemotherapy [61,62], enhancing destruction of tumor cells [63], and this favorable effect could become stronger in patients with highly immunogenic tumors, perhaps including the core basal group. Basal-like breast cancers have distinctive survival patterns, many relapses and deaths during the first 5 years after diagnosis, but fewer events after this period [32], indicating that basal-like breast cancers encompass both poor and good prognostic subgroups responding variably to conventional therapies. In our cohort, systemic treatment decisions were not randomized, making outcomes stratified by treatment difficult to interpret; nevertheless, an exploratory analysis suggests that pre-treatment CD8^+ ^lymphocyte infiltration is an independent favorable predictive indicator of good outcomes in basal-like cases treated with chemotherapy (HR = 0.29, 95% CI = 0.16 to 0.55, *P *< 0.001, *n *= 107) (Table S4 of Additional file 4). Our results indicate that efforts toward developing immuno-stimulative therapies might be best directed to the core basal group. The recognition of tumor-associated antigens by CD8^+ ^cells is a significant contributor to the detection and ultimate destruction of tumor cells [64]. Basal-like breast cancer could be particularly suitable for targeted immunotherapy. The lack of success of prior attempts at immunotherapy for breast cancer may be attributable, in part, to the lack of focus on appropriate breast cancer subtypes. A better understanding of the interaction between immune response, intrinsic subtype, AST, and patient outcome is critical to more effective and targeted clinical management for patients with breast cancer, especially those with basal-like breast tumors.

Studies on TILs in breast cancer have come to inconsistent conclusions. We believe that one of the underlying reasons could be inconsistency in defining and measuring TILs. Some research considered only the presence of peritumoral stromal lymphocytes [65,66], and many considered all T lymphocytes (which might include larger numbers of regulatory T cells that could in some cases reflect immune suppression instead of activation). In our study, specific immunohistochemistry was used with a mouse monoclonal anti-human CD8 antibody to detect cytotoxic effector CD8^+ ^TILs in intratumoral and stromal locations for each tumor tissue core. We evaluated the reliability of repeated scoring by the same scorer and between different scorers, and it was demonstrated that our visual CD8^+ ^TILs scoring was highly reliable (Figure S4 of Additional file 6). Analyses with intratumoral, stromal, and total CD8^+ ^TILs were conducted, and consistent results were obtained. We also did analyses using relapse-free survival as an outcome and obtained results similar to those using BCSS as the outcome (Figures S5 and S6 of Additional files 7 and 8 and Tables S5 to S7 of Additional file 4). Thus, we are confident that the identification and quantification of TILs and the assessment of the association of TILs with clinical outcome in breast cancer are reliable and valid in this study. One potential limitation of our methods is that TMAs may not adequately represent breast tumor heterogeneity. Several studies nevertheless have shown that findings from TMAs are consistent with those from full-face tissue sections [67,68]. Although we observed a trend to a favorable prognostic effect of CD8 TILs in the HER2^+^/ER^- ^subgroup (and this trend is consistent with a gene expression study [69]), the effect was not statistically significant in our univariate or multivariate analyses. Research with more power particularly for this subgroup needs to be done to draw a more definitive conclusion among HER2^+ ^cases. We were not able to measure changes in immune response induced by chemotherapy, as all of the tissue samples were collected before patients received systemic therapy. Further studies would need to be conducted to assess the interaction of TILs with chemotherapy, ideally in randomized trials.

## Conclusions

This study provides strong evidence that CD8^+ ^lymphocyte infiltration is an independent factor associated with improved survival in patients with breast cancer. The favorable prognostic effects of TILs occur mostly in the basal-like intrinsic subgroup.

## Abbreviations

5NP: five negative phenotype; AST: adjuvant systemic therapy; BCCA: British Columbia Cancer Agency; BCSS: breast cancer-specific survival; CI: confidence interval; CK: cytokeratin; EGFR: epidermal growth factor receptor; ER: estrogen receptor; HER2: human epidermal growth factor receptor-2; HR: hazard ratio; ICC: intraclass correlation coefficient; IQR: interquartile range; iTIL: intratumoral tumor-infiltrating lymphocyte; LVI: lymphovascular invasion; PR: progesterone receptor; sTIL: stromal tumor-infiltrating lymphocyte; Th: T helper; TIL: tumor-infiltrating lymphocyte; TMA: tissue microarray; TNP: triple-negative phenotype; tTIL: total CD8^+ ^tumor-infiltrating lymphocyte.

## Competing interests

The authors declare that they have no competing interests.

## Authors' contributions

SLi coordinated the study, analyzed data, and drafted the manuscript. JL advised on scoring and edited the manuscript. SLe assisted with statistical analyses. DG generated primary data. WDF provided the idea for the study, helped with data analysis, and edited the manuscript. TON organized the study, directed data generation and analysis, and edited the manuscript. All authors read and approved the final manuscript.

## Supplementary Material

Additional file 1 **Validation of the cutoff points of TILs**. The Supplemental method section explained how the receiver operating characteristic (ROC) analysis was used to validate the optimal cutoffs of TILs chosen from an independent study. To take into consideration that outcome variable, breast cancer specific survival, is a time to event endpoint, X-tile software was also used to validate the optimal cut-offs, and the same cutoff points of iTIL and sTIL were obtained as those from the ROC method. Click here for file

Additional file 2 **CD8+ TILs in breast cancer**. This image showed some examples of CD8+ iTIL and sTIL in a breast tumor sample (scale bar: 50 μm). Information with respect to availability of all of our CD8 staining images were provided in the figure legend. Click here for file

Additional file 3 **Distributions of CD8+ iTIL and sTIL in the whole cohort**. Histograms were used to show the distributions of CD8+ iTIL and sTIL in the whole study population. Values on the X-axis represent absolute counts of CD8+ iTIL (A) or sTIL (B) per tissue microarry core. Click here for file

Additional file 4 **Supplemental tables**. Table S1 showed the distributions of CD8+ sTIL and tTIL in relation to patient and tumor characteristics. Table S2 showed the hazard ratios (HRs) of sTIL and tTIL in the whole cohort with multivariate Cox regression analysis, adjusted by age at diagnosis, tumor grade and size, lymph node status, lymphovascular invasion, and intrinsic subtype. Table S3 showed the HRs of sTIL and tTIL in triple negative (TNP), core basal (CBP), and five negative (5NP) breast cancer intrinsic subgroups in multivariate analysis. Table S4 showed the HRs of iTIL, sTIL and tTIL in patients without adjuvant systemic therapy (AST) and with chemotherapy in multivariate analysis. Table S5 showed HRs of iTIL in the whole cohort with univariate and mulvariate analysis, using relapse-free survival (RFS) as the outcome variable. Tables S6 and S7 showed the HRs of iTIL in different intrinsic subgroups with multivariate Cox regression analysis using RFS as the outcome variable. Click here for file

Additional file 5 **Breast cancer specific survival (BCSS) by sTIL and tTIL in different breast cancer intrinsic subgroups**. Kaplan-Meier function survival analysis of association of TILs with BCSS: (A) sTIL in triple negative (TNP), (B) tTIL in TNP, (C) sTIL in core basal (CBP), (D) tTIL in CBP, (E) sTIL in five negative (5NP), and (F) tTIL in 5NP. Click here for file

Additional file 6 **Correlation of re-scoring of CD8+ TILs by the same and different pathologists**. The scatter plots demonstrated correlations of repeated scoring for 490 cases by the same pathologist for CD8+ iTIL (A) and sTIL (B), and re-scoring of CD8+ iTIL for 200 cases by two pathologists (C). Click here for file

Additional file 7 **Relapse-free survival (RFS) by iTIL among groups with different age and ER status**. Kaplan-Meier function survival analysis of association between iTIL and RFS in: (A) age < 50 year, (B) age ≥ 50 year; (C) ER-, and (D) ER+. Click here for file

Additional file 8 **Relapse-free survival (RFS) by iTIL in different breast cancer intrinsic subgroups**. Kaplan-Meier function survival analysis of association between iTIL and RFS in: (A) luminal A, (B) luminal B, (C) HER2+/ER-, (D) Triple negative, (E) core basal, and (F) five negative subgroups. Click here for file

## References

[B1] Tsuta K Ishii G Kim E Shiono S Nishiwaki Y Endoh Y Kodama T Nagai K Nagai K Primary lung adenocarcinoma with massive lymphocyte infiltration Am J Clin Pathol 2005 123 547 552 10.1309/APKQ4Q9D52GNLR8W 15743751

[B2] Canna K McArdle PA McMillan DC McNicol AM Smith GW McKee RF McArdle CS The relationship between tumour T-lymphocyte infiltration, the systemic inflammatory response and survival in patients undergoing curative resection for colorectal cancer Br J Cancer 2005 92 651 654 10.1038/sj.bjc.6602419 15700032 PMC2361875

[B3] Zhang L Conejo-Garcia JR Katsaros D Gimotty PA Massobrio M Regnani G Makrigiannakis A Gray H Schlienger K Liebman MN Rubin SC Coukos G Intratumoral T cells, recurrence, and survival in epithelial ovarian cancer N Engl J Med 2003 348 203 213 10.1056/NEJMoa020177 12529460

[B4] Clemente CG Mihm MC Jr Bufalino R Zurrida S Collini P Cascinelli N Prognostic value of tumor infiltrating lymphocytes in the vertical growth phase of primary cutaneous melanoma Cancer 1996 77 1303 1310 10.1002/(SICI)1097-0142(19960401)77:7<1303::AID-CNCR12>3.0.CO;2-5 8608507

[B5] Furihata M Ohtsuki Y Sonobe H Araki K Ogata T Toki T Ogoshi S Tamiya T Prognostic significance of simultaneous infiltration of HLA-DR-positive dendritic cells and tumor infiltrating lymphocytes into human esophageal carcinoma Tohoku J Exp Med 1993 169 187 195 10.1620/tjem.169.187 8248911

[B6] Jass JR Lymphocytic infiltration and survival in rectal cancer J Clin Pathol 1986 39 585 589 10.1136/jcp.39.6.585 3722412 PMC499954

[B7] Menard S Tomasic G Casalini P Balsari A Pilotti S Cascinelli N Salvadori B Colnaghi MI Rilke F Lymphoid infiltration as a prognostic variable for early-onset breast carcinomas Clin Cancer Res 1997 3 817 819 9815754

[B8] Nakano O Sato M Naito Y Suzuki K Orikasa S Aizawa M Suzuki Y Shintaku I Nagura H Ohtani H Proliferative activity of intratumoral CD8(+) T-lymphocytes as a prognostic factor in human renal cell carcinoma: clinicopathologic demonstration of antitumor immunity Cancer Res 2001 61 5132 5136 11431351

[B9] Schondorf T Engel H Lindemann C Kolhagen H von Rucker AA Mallmann P Cellular characteristics of peripheral blood lymphocytes and tumour-infiltrating lymphocytes in patients with gynaecological tumours Cancer Immunol Immunother 1997 44 88 96 10.1007/s002620050360 9177470 PMC11037785

[B10] Ben-Hur H Cohen O Schneider D Gurevich P Halperin R Bala U Mozes M Zusman I The role of lymphocytes and macrophages in human breast tumorigenesis: an immunohistochemical and morphometric study Anticancer Res 2002 22 1231 1238 12168931

[B11] Leong PP Mohammad R Ibrahim N Ithnin H Abdullah M Davis WC Seow HF Phenotyping of lymphocytes expressing regulatory and effector markers in infiltrating ductal carcinoma of the breast Immunol Lett 2006 102 229 236 10.1016/j.imlet.2005.09.006 16246429

[B12] Schillaci R Salatino M Cassataro J Proietti CJ Giambartolomei GH Rivas MA Carnevale RP Charreau EH Elizalde PV Immunization with murine breast cancer cells treated with antisense oligodeoxynucleotides to type I insulin-like growth factor receptor induced an antitumoral effect mediated by a CD8+ response involving Fas/Fas ligand cytotoxic pathway J Immunol 2006 176 3426 3437 16517711 10.4049/jimmunol.176.6.3426

[B13] Dobrzanski MJ Reome JB Hylind JC Rewers-Felkins KA CD8-mediated type 1 antitumor responses selectively modulate endogenous differentiated and nondifferentiated T cell localization, activation, and function in progressive breast cancer J Immunol 2006 177 8191 8201 17114496 10.4049/jimmunol.177.11.8191

[B14] Pages F Berger A Camus M Sanchez-Cabo F Costes A Molidor R Mlecnik B Kirilovsky A Nilsson M Damotte D Meatchi T Bruneval P Cugnenc PH Trajanoski Z Fridman WH Galon J Effector memory T cells, early metastasis, and survival in colorectal cancer N Engl J Med 2005 353 2654 2666 10.1056/NEJMoa051424 16371631

[B15] Lee AH Gillett CE Ryder K Fentiman IS Miles DW Millis RR Different patterns of inflammation and prognosis in invasive carcinoma of the breast Histopathology 2006 48 692 701 10.1111/j.1365-2559.2006.02410.x 16681685

[B16] Yakirevich E Izhak OB Rennert G Kovacs ZG Resnick MB Cytotoxic phenotype of tumor infiltrating lymphocytes in medullary carcinoma of the breast Mod Pathol 1999 12 1050 1056 10574602

[B17] Rakha EA Aleskandarany M El-Sayed ME Blamey RW Elston CW Ellis IO Lee AH The prognostic significance of inflammation and medullary histological type in invasive carcinoma of the breast Eur J Cancer 2009 45 1780 1787 10.1016/j.ejca.2009.02.014 19286369

[B18] Matkowski R Gisterek I Halon A Lacko A Szewczyk K Staszek U Pudelko M Szynglarewicz B Szelachowska J Zolnierek A Kornafel J The prognostic role of tumor-infiltrating CD4 and CD8 T lymphocytes in breast cancer Anticancer Res 2009 29 2445 2451 19596912

[B19] Carlomagno C Perrone F Lauria R de Laurentiis M Gallo C Morabito A Pettinato G Panico L Bellelli T Apicella A Prognostic significance of necrosis, elastosis, fibrosis and inflammatory cell reaction in operable breast cancer Oncology 1995 52 272 277 10.1159/000227472 7777238

[B20] Aaltomaa S Lipponen P Eskelinen M Kosma VM Marin S Alhava E Syrjanen K Lymphocyte infiltrates as a prognostic variable in female breast cancer Eur J Cancer 1992 28A 859 864 1524909 10.1016/0959-8049(92)90134-n

[B21] Camp BJ Dyhrman ST Memoli VA Mott LA Barth RJ Jr In situ cytokine production by breast cancer tumor-infiltrating lymphocytes Ann Surg Oncol 1996 3 176 184 10.1007/BF02305798 8646519

[B22] Liu F Lang R Zhao J Zhang X Pringle GA Fan Y Yin D Gu F Yao Z Fu L CD8(+) cytotoxic T cell and FOXP3(+) regulatory T cell infiltration in relation to breast cancer survival and molecular subtypes Breast Cancer Res Treat 2011 130 645 655 10.1007/s10549-011-1647-3 21717105

[B23] Mahmoud SM Paish EC Powe DG Macmillan RD Grainge MJ Lee AH Ellis IO Green AR Tumor-infiltrating CD8+ lymphocytes predict clinical outcome in breast cancer J Clin Oncol 2011 29 1949 1955 10.1200/JCO.2010.30.5037 21483002

[B24] Baker K Lachapelle J Zlobec I Bismar TA Terracciano L Foulkes WD Prognostic significance of CD8(+) T lymphocytes in breast cancer depends upon both oestrogen receptor status and histological grade Histopathology 2011 58 1107 1116 21707712 10.1111/j.1365-2559.2011.03846.x

[B25] Eden P Ritz C Rose C Ferno M Peterson C 'Good Old' clinical markers have similar power in breast cancer prognosis as microarray gene expression profilers Eur J Cancer 2004 40 1837 1841 10.1016/j.ejca.2004.02.025 15288284

[B26] Nimeus-Malmstrom E Ritz C Eden P Johnsson A Ohlsson M Strand C Ostberg G Ferno M Peterson C Gene expression profilers and conventional clinical markers to predict distant recurrences for premenopausal breast cancer patients after adjuvant chemotherapy Eur J Cancer 2006 42 2729 2737 10.1016/j.ejca.2006.06.031 17023159

[B27] Cleator S Ashworth A Molecular profiling of breast cancer: clinical implications Br J Cancer 2004 90 1120 1124 10.1038/sj.bjc.6601667 15026788 PMC2409657

[B28] Early Breast Cancer Trialists' Collaborative Group (EBCTCG) Effects of chemotherapy and hormonal therapy for early breast cancer on recurrence and 15-year survival: an overview of the randomised trials Lancet 2005 365 1687 1717 15894097 10.1016/S0140-6736(05)66544-0

[B29] Carey L Winer E Viale G Cameron D Gianni L Triple-negative breast cancer: disease entity or title of convenience? Nat Rev Clin Oncol 2010 7 683 692 10.1038/nrclinonc.2010.154 20877296

[B30] Bertucci F Finetti P Cervera N Esterni B Hermitte F Viens P Birnbaum D How basal are triple-negative breast cancers? Int J Cancer 2008 123 236 240 10.1002/ijc.23518 18398844

[B31] Cheang MC Voduc D Bajdik C Leung S McKinney S Chia SK Perou CM Nielsen TO Basal-like breast cancer defined by five biomarkers has superior prognostic value than triple-negative phenotype Clin Cancer Res 2008 14 1368 1376 10.1158/1078-0432.CCR-07-1658 18316557

[B32] Blows FM Driver KE Schmidt MK Broeks A van Leeuwen FE Wesseling J Cheang MC Gelmon K Nielsen TO Blomqvist C Heikkila P Heikkinen T Nevanlinna H Akslen LA Begin LR Foulkes WD Couch FJ Wang X Cafourek V Olson JE Baglietto L Giles GG Severi G McLean CA Southey MC Rakha E Green AR Ellis IO Sherman ME Lissowska J Subtyping of breast cancer by immunohistochemistry to investigate a relationship between subtype and short and long term survival: a collaborative analysis of data for 10,159 cases from 12 studies PLoS Med 2010 7 e1000279 10.1371/journal.pmed.1000279 20520800 PMC2876119

[B33] Bertucci F Finetti P Rougemont J Charafe-Jauffret E Cervera N Tarpin C Nguyen C Xerri L Houlgatte R Jacquemier J Viens P Birnbaum D Gene expression profiling identifies molecular subtypes of inflammatory breast cancer Cancer Res 2005 65 2170 2178 10.1158/0008-5472.CAN-04-4115 15781628

[B34] Bertucci F Finetti P Cervera N Charafe-Jauffret E Mamessier E Adelaide J Debono S Houvenaeghel G Maraninchi D Viens P Charpin C Jacquemier J Birnbaum D Gene expression profiling shows medullary breast cancer is a subgroup of basal breast cancers Cancer Res 2006 66 4636 4644 10.1158/0008-5472.CAN-06-0031 16651414

[B35] Mouawad R Spano JP Khayat D Lymphocyte infiltration in breast cancer: a key prognostic factor that should not be ignored J Clin Oncol 2011 29 1935 1936 10.1200/JCO.2011.35.4845 21482993

[B36] Qian BZ Pollard JW Macrophage diversity enhances tumor progression and metastasis Cell 2010 141 39 51 10.1016/j.cell.2010.03.014 20371344 PMC4994190

[B37] Cheang MC Treaba DO Speers CH Olivotto IA Bajdik CD Chia SK Goldstein LC Gelmon KA Huntsman D Gilks CB Nielsen TO Gown AM Immunohistochemical detection using the new rabbit monoclonal antibody SP1 of estrogen receptor in breast cancer is superior to mouse monoclonal antibody 1D5 in predicting survival J Clin Oncol 2006 24 5637 5644 10.1200/JCO.2005.05.4155 17116944

[B38] Lohrisch C Jackson J Jones A Mates D Olivotto IA Relationship between tumor location and relapse in 6,781 women with early invasive breast cancer J Clin Oncol 2000 18 2828 2835 10920130 10.1200/JCO.2000.18.15.2828

[B39] Liu S Chia SK Mehl E Leung S Rajput A Cheang MC Nielsen TO Progesterone receptor is a significant factor associated with clinical outcomes and effect of adjuvant tamoxifen therapy in breast cancer patients Breast Cancer Res Treat 2010 119 53 61 10.1007/s10549-009-0318-0 19205877

[B40] Chia S Norris B Speers C Cheang M Gilks B Gown AM Huntsman D Olivotto IA Nielsen TO Gelmon K Human epidermal growth factor receptor 2 overexpression as a prognostic factor in a large tissue microarray series of node-negative breast cancers J Clin Oncol 2008 26 5697 5704 10.1200/JCO.2007.15.8659 19001334

[B41] Nielsen TO Hsu FD Jensen K Cheang M Karaca G Hu Z Hernandez-Boussard T Livasy C Cowan D Dressler L Akslen LA Ragaz J Gown AM Gilks CB van de Rijn M Perou CM Immunohistochemical and clinical characterization of the basal-like subtype of invasive breast carcinoma Clin Cancer Res 2004 10 5367 5374 10.1158/1078-0432.CCR-04-0220 15328174

[B42] Cheang MC Chia SK Voduc D Gao D Leung S Snider J Watson M Davies S Bernard PS Parker JS Perou CM Ellis MJ Nielsen TO Ki67 index, HER2 status, and prognosis of patients with luminal B breast cancer J Natl Cancer Inst 2009 101 736 750 10.1093/jnci/djp082 19436038 PMC2684553

[B43] Gatza ML Lucas JE Barry WT Kim JW Wang Q Crawford MD Datto MB Kelley M Mathey-Prevot B Potti A Nevins JR A pathway-based classification of human breast cancer Proc Natl Acad Sci USA 2010 107 6994 6999 10.1073/pnas.0912708107 20335537 PMC2872436

[B44] Teschendorff AE Miremadi A Pinder SE Ellis IO Caldas C An immune response gene expression module identifies a good prognosis subtype in estrogen receptor negative breast cancer Genome Biol 2007 8 R157 10.1186/gb-2007-8-8-r157 17683518 PMC2374988

[B45] Teschendorff AE Caldas C A robust classifier of high predictive value to identify good prognosis patients in ER-negative breast cancer Breast Cancer Res 2008 10 R73 10.1186/bcr2138 18755024 PMC2575547

[B46] Reyal F van Vliet MH Armstrong NJ Horlings HM de Visser KE Kok M Teschendorff AE Mook S van't Veer L Caldas C Salmon RJ van de Vijver MJ Wessels LF A comprehensive analysis of prognostic signatures reveals the high predictive capacity of the proliferation, immune response and RNA splicing modules in breast cancer Breast Cancer Res 2008 10 R93 10.1186/bcr2192 19014521 PMC2656909

[B47] Schmidt M Bohm D von Torne C Steiner E Puhl A Pilch H Lehr HA Hengstler JG Kolbl H Gehrmann M The humoral immune system has a key prognostic impact in node-negative breast cancer Cancer Res 2008 68 5405 5413 10.1158/0008-5472.CAN-07-5206 18593943

[B48] Desmedt C Haibe-Kains B Wirapati P Buyse M Larsimont D Bontempi G Delorenzi M Piccart M Sotiriou C Biological processes associated with breast cancer clinical outcome depend on the molecular subtypes Clin Cancer Res 2008 14 5158 5165 10.1158/1078-0432.CCR-07-4756 18698033

[B49] Calabro A Beissbarth T Kuner R Stojanov M Benner A Asslaber M Ploner F Zatloukal K Samonigg H Poustka A Sultmann H Effects of infiltrating lymphocytes and estrogen receptor on gene expression and prognosis in breast cancer Breast Cancer Res Treat 2009 116 69 77 10.1007/s10549-008-0105-3 18592372

[B50] Kreike B van Kouwenhove M Horlings H Weigelt B Peterse H Bartelink H van de Vijver MJ Gene expression profiling and histopathological characterization of triple-negative/basal-like breast carcinomas Breast Cancer Res 2007 9 R65 10.1186/bcr1771 17910759 PMC2242660

[B51] Sabatier R Finetti P Cervera N Lambaudie E Esterni B Mamessier E Tallet A Chabannon C Extra JM Jacquemier J Viens P Birnbaum D Bertucci F A gene expression signature identifies two prognostic subgroups of basal breast cancer Breast Cancer Res Treat 2011 126 407 420 10.1007/s10549-010-0897-9 20490655

[B52] Sabatier R Finetti P Mamessier E Raynaud S Cervera N Lambaudie E Jacquemier J Viens P Birnbaum D Bertucci F Kinome expression profiling and prognosis of basal breast cancers Mol Cancer 2011 10 86 10.1186/1476-4598-10-86 21777462 PMC3156788

[B53] DeNardo DG Barreto JB Andreu P Vasquez L Tawfik D Kolhatkar N Coussens LM CD4(+) T cells regulate pulmonary metastasis of mammary carcinomas by enhancing protumor properties of macrophages Cancer Cell 2009 16 91 102 10.1016/j.ccr.2009.06.018 19647220 PMC2778576

[B54] Teschendorff AE Gomez S Arenas A El-Ashry D Schmidt M Gehrmann M Caldas C Improved prognostic classification of breast cancer defined by antagonistic activation patterns of immune response pathway modules BMC Cancer 2010 10 604 10.1186/1471-2407-10-604 21050467 PMC2991308

[B55] Whitford P Mallon EA George WD Campbell AM Flow cytometric analysis of tumour infiltrating lymphocytes in breast cancer Br J Cancer 1990 62 971 975 10.1038/bjc.1990.419 2124138 PMC1971553

[B56] Georgiannos SN Renaut A Goode AW Sheaff M The immunophenotype and activation status of the lymphocytic infiltrate in human breast cancers, the role of the major histocompatibility complex in cell-mediated immune mechanisms, and their association with prognostic indicators Surgery 2003 134 827 834 10.1016/S0039-6060(03)00292-7 14639362

[B57] Smyth MJ Dunn GP Schreiber RD Cancer immunosurveillance and immunoediting: the roles of immunity in suppressing tumor development and shaping tumor immunogenicity Adv Immunol 2006 90 1 50 16730260 10.1016/S0065-2776(06)90001-7

[B58] Pedersen AE Stryhn A Justesen S Harndahl M Rasmussen S Donskov F Claesson MH Pedersen JW Wandall HH Svane IM Buus S Wildtype p53-specific antibody and T-cell responses in cancer patients J Immunother 2011 34 629 640 10.1097/CJI.0b013e3182281381 21989411

[B59] Svane IM Pedersen AE Johansen JS Johnsen HE Nielsen D Kamby C Ottesen S Balslev E Gaarsdal E Nikolajsen K Claesson MH Vaccination with p53 peptide-pulsed dendritic cells is associated with disease stabilization in patients with p53 expressing advanced breast cancer; monitoring of serum YKL-40 and IL-6 as response biomarkers Cancer Immunol Immunother 2007 56 1485 1499 10.1007/s00262-007-0293-4 17285289 PMC11030002

[B60] Hansen MH Nielsen H Ditzel HJ The tumor-infiltrating B cell response in medullary breast cancer is oligoclonal and directed against the autoantigen actin exposed on the surface of apoptotic cancer cells Proc Natl Acad Sci USA 2001 98 12659 12664 10.1073/pnas.171460798 11606714 PMC60110

[B61] Apetoh L Tesniere A Ghiringhelli F Kroemer G Zitvogel L Molecular interactions between dying tumor cells and the innate immune system determine the efficacy of conventional anticancer therapies Cancer Res 2008 68 4026 4030 10.1158/0008-5472.CAN-08-0427 18519658

[B62] Zitvogel L Apetoh L Ghiringhelli F Kroemer G Immunological aspects of cancer chemotherapy Nat Rev Immunol 2008 8 59 73 10.1038/nri2216 18097448

[B63] Zitvogel L Apetoh L Ghiringhelli F Andre F Tesniere A Kroemer G The anticancer immune response: indispensable for therapeutic success? J Clin Invest 2008 118 1991 2001 10.1172/JCI35180 18523649 PMC2396905

[B64] Del Campo AB Carretero J Aptsiauri N Garrido F Targeting HLA class I expression to increase tumor immunogenicity Tissue Antigens 2012 79 147 154 10.1111/j.1399-0039.2011.01831.x 22309256

[B65] Putti TC El-Rehim DM Rakha EA Paish CE Lee AH Pinder SE Ellis IO Estrogen receptor-negative breast carcinomas: a review of morphology and immunophenotypical analysis Mod Pathol 2005 18 26 35 10.1038/modpathol.3800255 15332092

[B66] Wernicke M Roitman P Manfre D Stern R Breast cancer and the stromal factor. The 'prometastatic healing process' hypothesis Medicina (B Aires) 2011 71 15 21 21296715

[B67] Schraml P Kononen J Bubendorf L Moch H Bissig H Nocito A Mihatsch MJ Kallioniemi OP Sauter G Tissue microarrays for gene amplification surveys in many different tumor types Clin Cancer Res 1999 5 1966 1975 10473073

[B68] Torhorst J Bucher C Kononen J Haas P Zuber M Kochli OR Mross F Dieterich H Moch H Mihatsch M Kallioniemi OP Sauter G Tissue microarrays for rapid linking of molecular changes to clinical endpoints Am J Pathol 2001 159 2249 2256 10.1016/S0002-9440(10)63075-1 11733374 PMC1850582

[B69] Alexe G Dalgin GS Scanfeld D Tamayo P Mesirov JP DeLisi C Harris L Barnard N Martel M Levine AJ Ganesan S Bhanot G High expression of lymphocyte-associated genes in node-negative HER2+ breast cancers correlates with lower recurrence rates Cancer Res 2007 67 10669 10676 10.1158/0008-5472.CAN-07-0539 18006808

