# Supplementary material for: CD8+ lymphocyte infiltration is an independent favorable prognostic indicator in basal-like breast cancer
Source: Breast Cancer Res. 2012 Mar 15;14(2):R48. doi: 10.1186/bcr3148 (PMC3446382; doi:10.1186/bcr3148)
Supplement: Additional file 1 — Validation of the cutoff points of TILs. The Supplemental method section explained how the receiver operating characteristic (ROC) analysis was used to validate the optimal cutoffs of TILs chosen from an independent study. To take into consideration that outcome variable, breast cancer specific survival, is a time to event endpoint, X-tile software was also used to validate the optimal cut-offs, and the same cutoff points of iTIL and sTIL were obtained as those from the ROC method. [file bcr3148-S1.PDF]

**Supplemental method**

**Validation of the cutoff points of TILs**

We chose, *a priori*, to use the optimal cutoff points of TILs developed from an independent study (Baker Histopathology 2011 1107-1116). However, the large size and detailed outcome data allowed us the opportunity to confirm the validity of the selected cutoff points for iTIL and sTIL using receiver operating characteristic (ROC) curve analysis (Zlobec British Journal of Cancer 2007 793-800). In brief, at each score, the sensitivity and specificity for the outcome of 10-year breast cancer specific survival were plotted thus generating an ROC curve. The score resulting in both maximum sensitivity and spedificity was selected as the optimal cutoff point. To evaluate if ROC was a reproducible method for selecting the cutoff points, a training set comprised of randomly selected cases (50% of the whole cohort) was used to select an optimal cutoff which was then tested on the independent left-out test set. Youden Index (sensitivity + specificity -1) was used to assess if the selected cutoff is valid in the test set. This procedure was repeated for 100 times to ensure robustness. The repeated training-test analysis showed that the optimal cutoffs were 1 (≥1 vs. 0) for iTIL (Interquartile Range, IQR = 1-1) and 3 (≥3 vs. <3) for sTIL (IQR = 1-3). The means of Youden Index in the test set for different cut-offs from the training set were shown in the table below.

Cut-offs of iTIL and sTIL from training set and the corresponding Youden Index in test set

| Cut-off from training set | iTIL | | sTIL | |
|---|---|---|---|---|
| | number of runs | Mean of Youden Index from test set (95% CI) | number of runs | Mean of Youden Index from test set (95% CI) |
| 1 | **89** | **0.024 (-0.008-0.053)** | 43 | 0.040 (0.015-0.068) |
| 2 | 3 | 0.004 (-0.003-NA) | 4 | 0.011 (-0.021-0.038) |
| 3 | 0 | NA | **53** | **0.045 (0.016-0.049)** |

To take into consideration that BCSS is a time to event endpoint, X-tile software version 3.6.1(Yale University School of Medicine, New Haven, CT) was also used to validate the optimal cut-offs, and the same cutoff points of iTIL and sTIL were obtained as those from the ROC method.
