# Supplementary material for: CD8+ lymphocyte infiltration is an independent favorable prognostic indicator in basal-like breast cancer
Source: Breast Cancer Res. 2012 Mar 15;14(2):R48. doi: 10.1186/bcr3148 (PMC3446382; doi:10.1186/bcr3148)
Supplement: Additional file 2 — CD8+ TILs in breast cancer. This image showed some examples of CD8+ iTIL and sTIL in a breast tumor sample (scale bar: 50 μm). Information with respect to availability of all of our CD8 staining images were provided in the figure legend. [file bcr3148-S2.PDF]

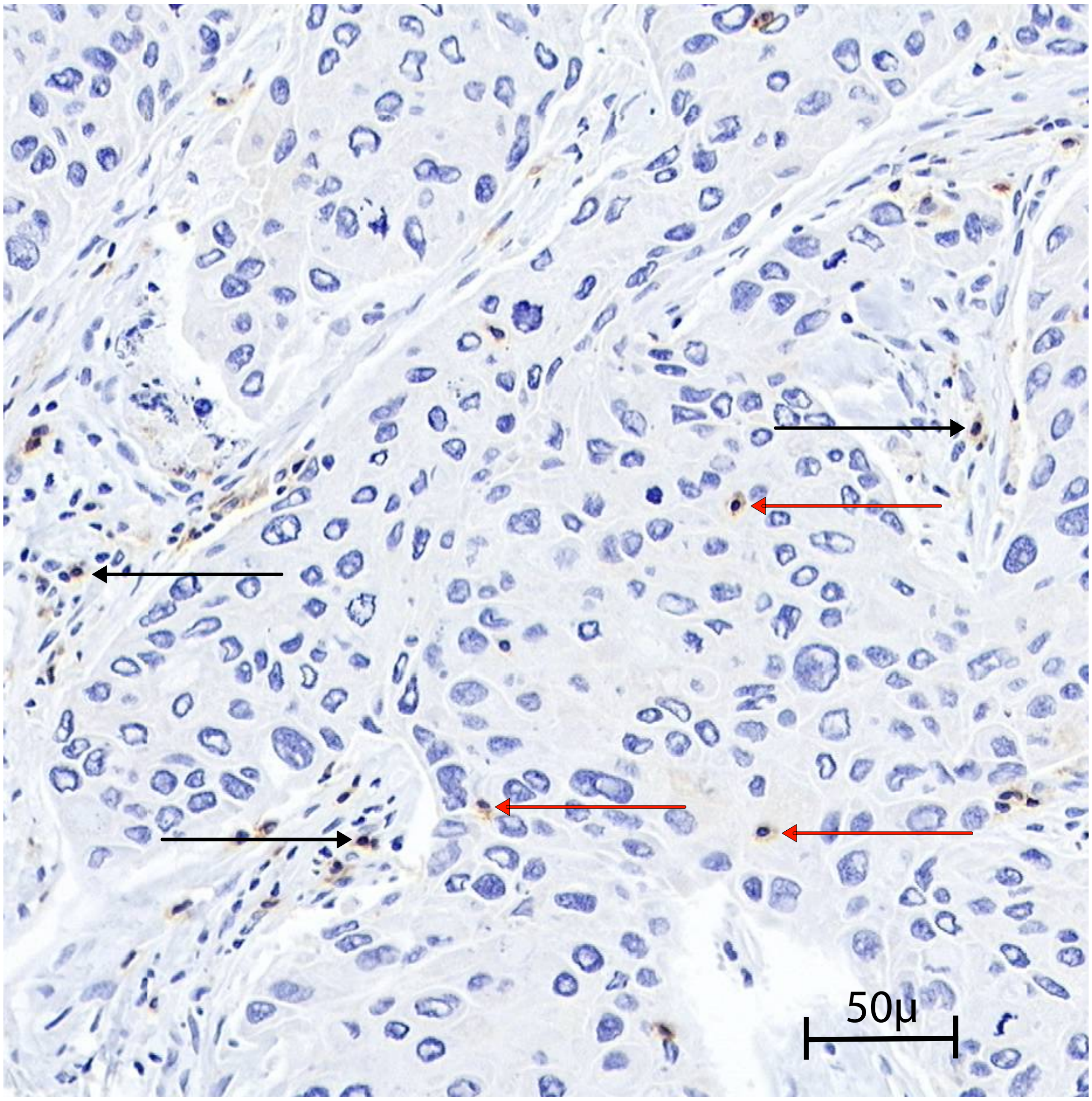

Figure S1. CD8+ TILs in breast cancer. Red arrows indicate examples of intratumoral CD8+ cells (iTIL), and black arrows show examples of stromal CD8+ cells (sTIL). All of our CD8 staining images are available at http://www.gpecimage.ubc.ca.
