# Supplementary material for: CD8+ lymphocyte infiltration is an independent favorable prognostic indicator in basal-like breast cancer
Source: Breast Cancer Res. 2012 Mar 15;14(2):R48. doi: 10.1186/bcr3148 (PMC3446382; doi:10.1186/bcr3148)
Supplement: Additional file 3 — Distributions of CD8+ iTIL and sTIL in the whole cohort. Histograms were used to show the distributions of CD8+ iTIL and sTIL in the whole study population. Values on the X-axis represent absolute counts of CD8+ iTIL (A) or sTIL (B) per tissue microarry core. [file bcr3148-S3.PDF]

A

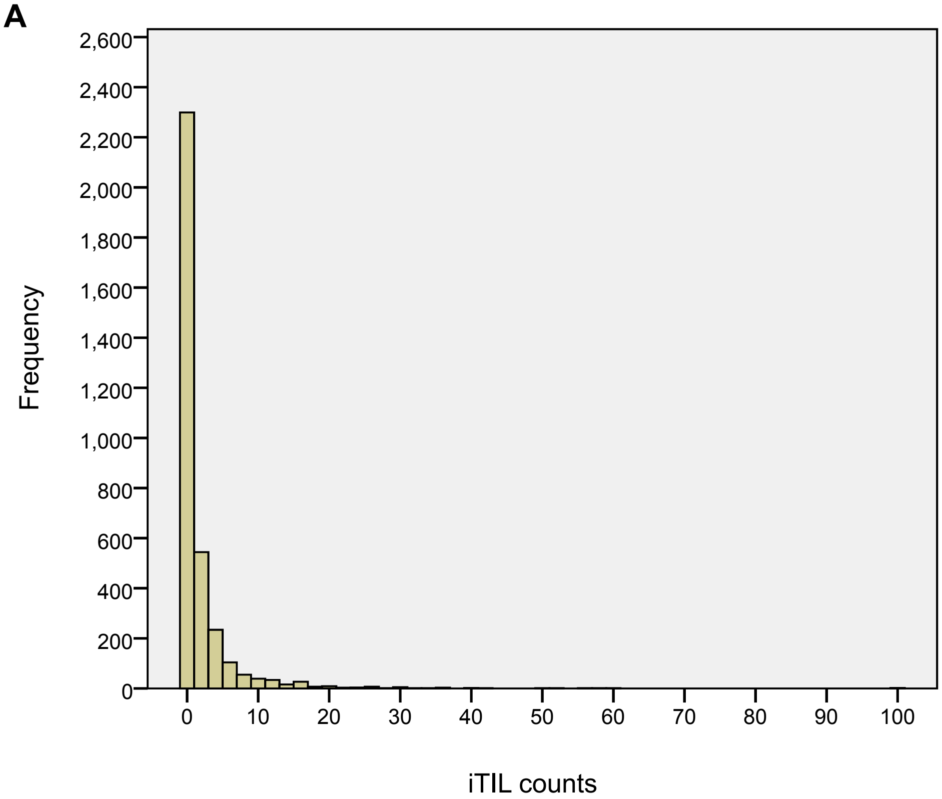

B

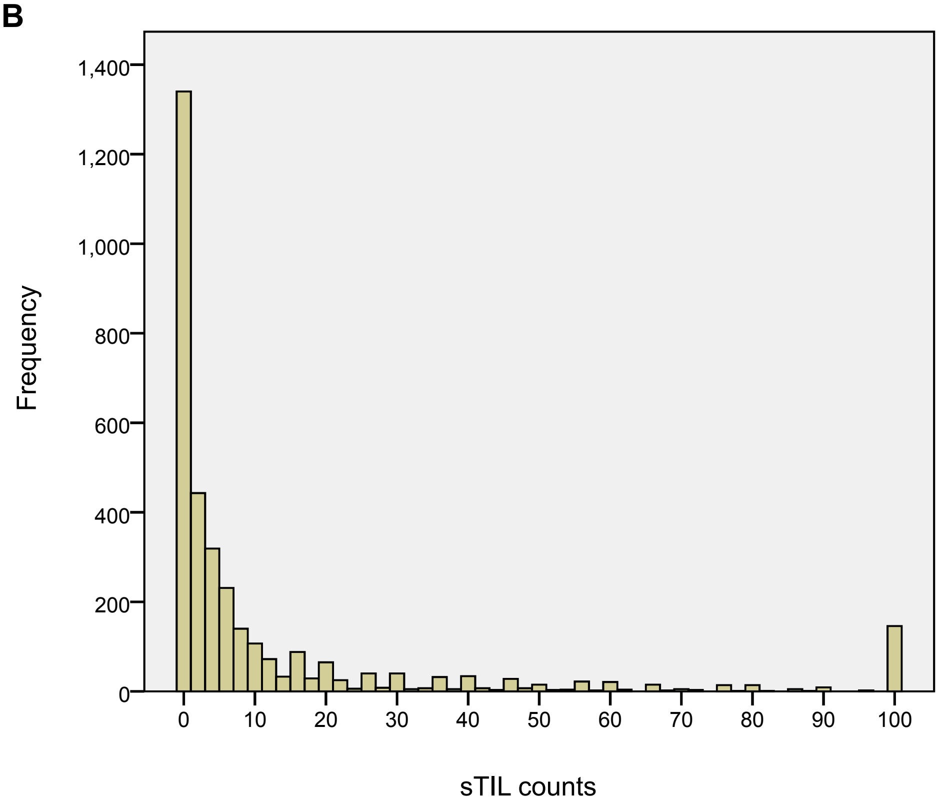

Figure S2. Distributions of CD8+ iTIL (A), and sTIL (B) in the whole cohort. Values represent absolute counts of CD8+ lymphocytes touching cancer cells (iTIL) or in adjacent stroma (sTIL) per tissue microarray core.
