# Supplementary material for: CD8+ lymphocyte infiltration is an independent favorable prognostic indicator in basal-like breast cancer
Source: Breast Cancer Res. 2012 Mar 15;14(2):R48. doi: 10.1186/bcr3148 (PMC3446382; doi:10.1186/bcr3148)
Supplement: Additional file 4 — Supplemental tables. Table S1 showed the distributions of CD8+ sTIL and tTIL in relation to patient and tumor characteristics. Table S2 showed the hazard ratios (HRs) of sTIL and tTIL in the whole cohort with multivariate Cox regression analysis, adjusted by age at diagnosis, tumor grade and size, lymph node status, lymphovascular invasion, and intrinsic subtype. Table S3 showed the HRs of sTIL and tTIL in triple negative (TNP), core basal (CBP), and five negative (5NP) breast cancer intrinsic subgroups in multivariate analysis. Table S4 showed the HRs of iTIL, sTIL and tTIL in patients without adjuvant systemic therapy (AST) and with chemotherapy in multivariate analysis. Table S5 showed HRs of iTIL in the whole cohort with univariate and mulvariate analysis, using relapse-free survival (RFS) as the outcome variable. Tables S6 and S7 showed the HRs of iTIL in different intrinsic subgroups with multivariate Cox regression analysis using RFS as the outcome variable. [file bcr3148-S4.PDF]

Supplemental tables

Table S1. Clinico-pathologic characteristics and distribution of sTIL and tTIL in the study population

Characteristics	No. patients (%)	sTILs (≥ 3)		tTILs (≥ 2)	
		%	p-value	%	p-value
**Age**			<0.001		<0.001
< 40	294 (7.4)	58.7 (148/252)		62.3 (147/236)	
40-49	844 (21.1)	54.3 (395/728)		62.1 (432/696)	
50-65	1,425 (35.7)	47.1 (567/1203)		55.0 (640/1164)	
> 65	1,429 (35.8)	41.8 (510/1220)		50.3 (594/1182)	
**Grade**			<0.001		<0.001
1	209 (5.2)	38.4 (63/164)		45.9 (73/159)	
2	1,563 (39.2)	42.0 (563/1342)		51.4 (679/1321)	
3	2,040 (51.1)	53.0 (929/1754)		59.6 (991/1663)	
unknown	180 (4.5)				
**Tumor size (cm)**			0.022		0.038
≤ 2	2,078 (52.1)	45.2 (800/1768)		53.8 (924/1717)	
> 2-5	1,667 (41.8)	49.8 (721/1449)		56.1 (776/1383)	
> 5	221 (5.5)	51.5 (87/169)		63.6 (105/165)	
unknown	26 (0.6)				
**Nodal status**			0.022		0.003
Negative	2,265 (56.7)	45.9 (877/1911)		53.0 (979/1847)	
Positive	1,719 (43.1)	49.9 (740/1484)		58.3 (830/1423)	
unknown	8 (0.2)				
**LVI**			0.181		0.117
Negative	2,106 (52.8)	46.3 (820/1770)		53.9 (921/1708)	
Positive	1,710 (42.8)	48.7 (727/1492)		56.7 (815/1437)	
Unknown	176 (4.4)				
**Histology**			<0.001		<0.001
Medullary	66 (1.7)	86.3 (44/51)		86.8 (33/38)	
Non-medullary	3926 (98.3)	47.0 (1576/3352)		54.9 (1780/3240)	
**AJCC stage**			0.017		0.015
I	1,393 (34.9)	44.3 (519/1172)		52.2 (597/1144)	
II	2,255 (56.5)	49.3 (965/1959)		56.7 (1061/1871)	
III	317 (7.9)	50.4 (127/252)		60.2 (148/246)	
Unknown/missing	27 (0.7)				
**AST**			<0.001		<0.001
No AST	1,676 (42.0)	45.7 (650/1422)		53.1 (735/1384)	
Tamoxifen only	1,276 (32.0)	42.6 (467/1097)		52.0 (550/1057)	
Chemotherapy only	727 (18.2)	55.9 (346/619)		62.6 (366/585)	
Tamoxifen+Chemo	297 (7.4)	59.2 (148/250)		64.1 (152/237)	
Other	16 (0.4)	60.0 (9/15)		66.7 (10/15)	
**ER**			<0.001		<0.001
Negative	1,200 (30.1)	54.6 (506/927)		60.5 (522/863)	
Positive	2,769 (69.1)	45.1 (1107/2456)		53.6 (1283/2395)	
Unknown	31 (0.8)				
**HER2**			<0.001		<0.001
Negative	3,316 (83.1)	45.5 (1319/2902)		53.4 (1947/2805)	
Positive	498 (12.5)	62.8 (279/444)		69.1 (289/418)	
Unknown	178 (4.4)				

**Subtype**			<0.001		<0.001
Luminal A	1,518 (38.0)	40.2 (560/1392)		49.5 (679/1372)	
Luminal B	829 (20.8)	51.1 (395/773)		59.1 (437/739)	
Luminal/HER2	224 (5.6)	62.6 (129/206)		67.2 (131/195)	
HER2+/ER-	250 (6.3)	63.9 (145/227)		71.8 (153/213)	
TNP	630 (15.8)	55.1 (295/535)		59.0 (291/493)	
Core basal	330 (8.3)	58.0 (178/307)		63.3 (179/283)	
5NP	162 (4.1)	54.9 (78/142)		56.5 (74/131)	
Not assignable	138 (3.4)	45.3 (39/86)		48.1 (38/79)	
Other	541 (13.5)	35.6 (96/270)		45.9 (122/266)	
**Total**	**3,992 (100)**	**47.6 (1620/3403)**		**55.3 (1813/3278)**	

Table S2. Hazards for breast cancer specific survival in the whole cohort with multivariate analysis

**Variable**	**sTIL (≥3 vs. <3)**<br>**HR (95% CI)**	**tTIL (≥2 vs. <2)**<br>**HR (95% CI)**
Age (≥50 vs. < 50)	1.00 (0.87 - 1.15)	0.99 (0.86 - 1.15)
Grade (3 vs. 1 + 2)	1.55 (1.34 - 1.80)	1.53 (1.32 - 1.78)
Tumor size (> 2 cm vs. ≤ 2 cm)	1.59 (1.38 - 1.83)	1.61 (1.40 - 1.86)
Nodal Status (Positive vs. negative)	2.04 (1.75 - 2.38)	2.08 (1.78 - 2.43)
LVI (Positive vs. negative)	1.31 (1.12 - 1.53)	1.32 (1.13 - 1.54)
Subtype		
Luminal B vs. luminal A	1.73 (1.45 - 2.07)	1.74 (1.45 - 2.08)
HER2/ER- vs. luminal A	2.55 (2.01 - 3.24)	2.66 (2.09 - 3.39)
Core basal-like vs. luminal A	1.95 (1.53 - 2.48)	2.11 (1.65 - 2.69)
5NP vs. luminal A	1.50 (1.13 - 1.99)	1.57 (1.18 - 2.10)
TILs	0.85 (0.74 - 0.97)	0.83 (0.73 - 0.95)

Table S3. Hazards for breast cancer specific survival with multivariate analysis in TNP, CBP and 5NP subgroups

**Variable**	**sTIL (≥3 vs. <3)**			**tTIL (≥2 vs. <2)**		
	**HR in TNP**	**HR in CBP**	**HR in 5NP**	**HR in TNP**	**HR in CBP**	**HR in 5NP**
Age<br>(≥ 50 vs. < 50)	0.88<br>(0.64-1.20)	0.87<br>(0.58-1.29)	1.07<br>(0.54-2.11)	0.87<br>(0.63-1.20)	0.84<br>(0.56-1.26)	1.26<br>(0.64-2.61)
Grade<br>(3 vs. 1 + 2)	1.70<br>(1.09-2.65)	1.40<br>(0.73-2.69)	1.98<br>(0.79-4.96)	1.65<br>(1.06-2.58)	1.33<br>(0.69-2.57)	2.02<br>(0.81-5.04)
Tumor size<br>(> 2 cm vs. ≤ 2 cm)	1.57<br>(1.13-2.17)	1.52<br>(1.01-2.27)	1.50<br>(0.72-3.14)	1.60<br>(1.14-2.22)	1.61<br>(1.07-2.43)	1.34<br>(0.61-2.91)
Nodal Status<br>(Positive vs. negative)	2.05<br>(1.43-2.84)	2.02<br>(1.31-3.10)	1.67<br>(0.76-3.65)	2.13<br>(1.50-3.02)	2.02<br>(1.31-3.14)	1.96<br>(0.87-4.39)
LVI<br>(Positive vs. negative)	1.63<br>(1.15-2.32)	1.55<br>(1.00-2.40)	3.08<br>(1.26-7.54)	1.69<br>(1.18-2.43)	1.58<br>(1.01-2.47)	3.96<br>(1.47-10.64)
TILs	0.52<br>(0.38-0.71)	0.48<br>(0.32-0.73)	0.82<br>(0.38-1.72)	0.55<br>(0.40-0.76)	0.44<br>(0.29-0.67)	1.11<br>(0.51-2.41)

Table S4. Hazards ratio for breast cancer specific survival with multivariate analysis in core basal-like subgroup with and without chemotherapy

Variable	iTIL (≥1 vs. 0)		sTIL (≥3 vs. <3)		tTIL (≥2 vs. <2)	
	no AST	chemotherapy	no AST	chemotherapy	no AST	chemotherapy
Age (≥ 50 vs. < 50)	0.77 (0.36-1.65)	1.23 (0.63-2.46)	0.85 (0.40-1.80)	1.11 (0.56-2.20)	0.88 (0.41-1.90)	1.12 (0.56-2.25)
Grade (3 vs. 1 + 2)	3.69 (0.87-15.59)	0.73 (0.32-1.67)	3.37 (0.80-14.31)	0.62 (0.27-1.41)	3.34 (0.79-14.16)	0.59 (0.26-1.36)
Tumor size (> 2 cm vs. ≤ 2 cm)	2.31 (1.21-4.41)	1.95 (1.03-3.68)	2.00 (1.05-3.79)	1.51 (0.81-2.81)	1.86 (0.97-3.55)	1.83 (0.96-3.51)
Nodal Status (Positive vs. negative)	3.28 (1.51-7.12)	1.24 (0.64-2.41)	2.85 (1.33-6.09)	1.08 (0.56-2.07)	2.89 (1.34-6.25)	1.11 (0.58-2.14)
LVI (Positive vs. negative)	2.10 (1.03-4.29)	1.27 (0.66-2.43)	2.07 (1.02-4.19)	1.32 (0.68-2.55)	1.92 (0.93-3.96)	1.44 (0.75-2.78)
TILs	0.44 (0.22-0.86)	0.29 (0.16-0.55)	0.64 (0.33-1.24)	0.35 (0.20-0.63)	0.59 (0.30-1.14)	0.31 (0.17-0.56)

Table S5. Hazards for relapse-free survival in the whole cohort with univariate and multivariate analysis

Variable	Univariate analysis HR (95% CI)	Multivariate analysis HR (95% CI) (n=3145)
Age (≥ 50 vs. < 50)	0.80 (0.72 - 0.89)	0.88 (0.77 - 0.99)
Grade (3 vs. 1 + 2)	1.79 (1.60 - 1.99)	1.40 (1.23 - 1.59)
Tumor size (> 2 cm vs. ≤ 2 cm)	1.81 (1.63 - 2.00)	1.39 (1.24 - 1.57)
Nodal Status (Positive vs. negative)	2.22 (2.00 - 2.47)	1.76 (1.54 - 2.01)
LVI (Positive vs. negative)	1.86 (1.68 - 2.07)	1.18 (1.03 - 1.35)
Subtype		
Luminal B vs. luminal A	1.70 (1.48 - 1.96)	1.49 (1.28 - 1.74)
HER2/ER- vs. luminal A	2.35 (1.93 - 2.86)	2.08 (1.68 - 2.58)
Core basal vs. luminal A	1.79 (1.48 - 2.16)	1.58 (1.28 - 1.96)
5NP vs. luminal A	1.25 (1.01 - 1.55)	1.13 (0.88 - 1.46)
iTIL (≥ 1 vs. 0)	0.99 (0.88 - 1.12)	0.81 (0.71 - 0.92)

Table S6. Hazards for relapse-free survival with multivariate analysis in the luminal A, luminal B, and HER2+/ER- intrinsic subgroup

Variable	HR in LumA n = 1276	HR in LumB n = 715	HR in HER2+/ER- n = 216
Age (≥ 50 vs. < 50)	0.93 (0.74 - 1.16)	0.88 (0.70 -1.13)	1.10 (0.76 - 1.62)
Grade (3 vs. 1 + 2)	1.46 (1.19 - 1.79)	1.18 (0.94 - 1.49)	1.80 (1.09 - 2.98)
Tumor size (> 2 cm vs. ≤ 2 cm)	1.33 (1.08 - 1.63)	1.40 (1.10 - 1.79)	1.51 (1.02 - 2.34)
Nodal Status (Positive vs. negative)	1.77 (1.41 - 2.23)	1.56 (1.21 - 2.01)	1.55 (0.99 - 2.43)
LVI (Positive vs. negative)	1.07 (0.85 - 1.34)	1.21 (0.93 - 1.56)	1.23 (0.79 - 1.91)
iTIL (≥ 1 vs. 0)	1.03 (0.82 - 1.29)	0.90 (0.71 - 1.14)	0.75 (0.51 - 1.11)

Table S7. Hazards for relapse-free survival with multivariate analysis in TNP, core basal and 5NP groups

Variable	HR in TNP n = 496	HR in core basal n = 287	HR in 5NP n = 130
Age (≥ 50 vs. < 50)	0.90 (0.68 - 1.20)	0.94 (0.65 - 1.34)	1.04 (0.55 - 1.94)
Grade (3 vs. 1 + 2)	1.72 (1.15 - 2.60)	1.69 (0.91 - 3.16)	1.57 (0.71 - 3.47)
Tumor size (> 2 cm vs. ≤ 2 cm)	1.57 (1.17 - 2.17)	1.66 (1.14 - 2.41)	1.40 (0.72 - 2.73)
Nodal Status (Positive vs. negative)	1.68 (1.23 - 2.29)	1.81 (1.22 - 2.70)	1.23 (0.63 - 2.42)
LVI (Positive vs. negative)	1.41 (1.02 - 1.94)	1.33 (0.90 - 1.99)	3.31 (1.48 - 7.38)
iTIL (≥ 1 vs. 0)	0.53 (0.39 - 0.72)	0.41 (0.28 - 0.61)	0.98 (0.50 - 1.89)
