# Supplementary material for: CD8+ lymphocyte infiltration is an independent favorable prognostic indicator in basal-like breast cancer
Source: Breast Cancer Res. 2012 Mar 15;14(2):R48. doi: 10.1186/bcr3148 (PMC3446382; doi:10.1186/bcr3148)
Supplement: Additional file 5 — Breast cancer specific survival (BCSS) by sTIL and tTIL in different breast cancer intrinsic subgroups. Kaplan-Meier function survival analysis of association of TILs with BCSS: (A) sTIL in triple negative (TNP), (B) tTIL in TNP, (C) sTIL in core basal (CBP), (D) tTIL in CBP, (E) sTIL in five negative (5NP), and (F) tTIL in 5NP. [file bcr3148-S5.PDF]

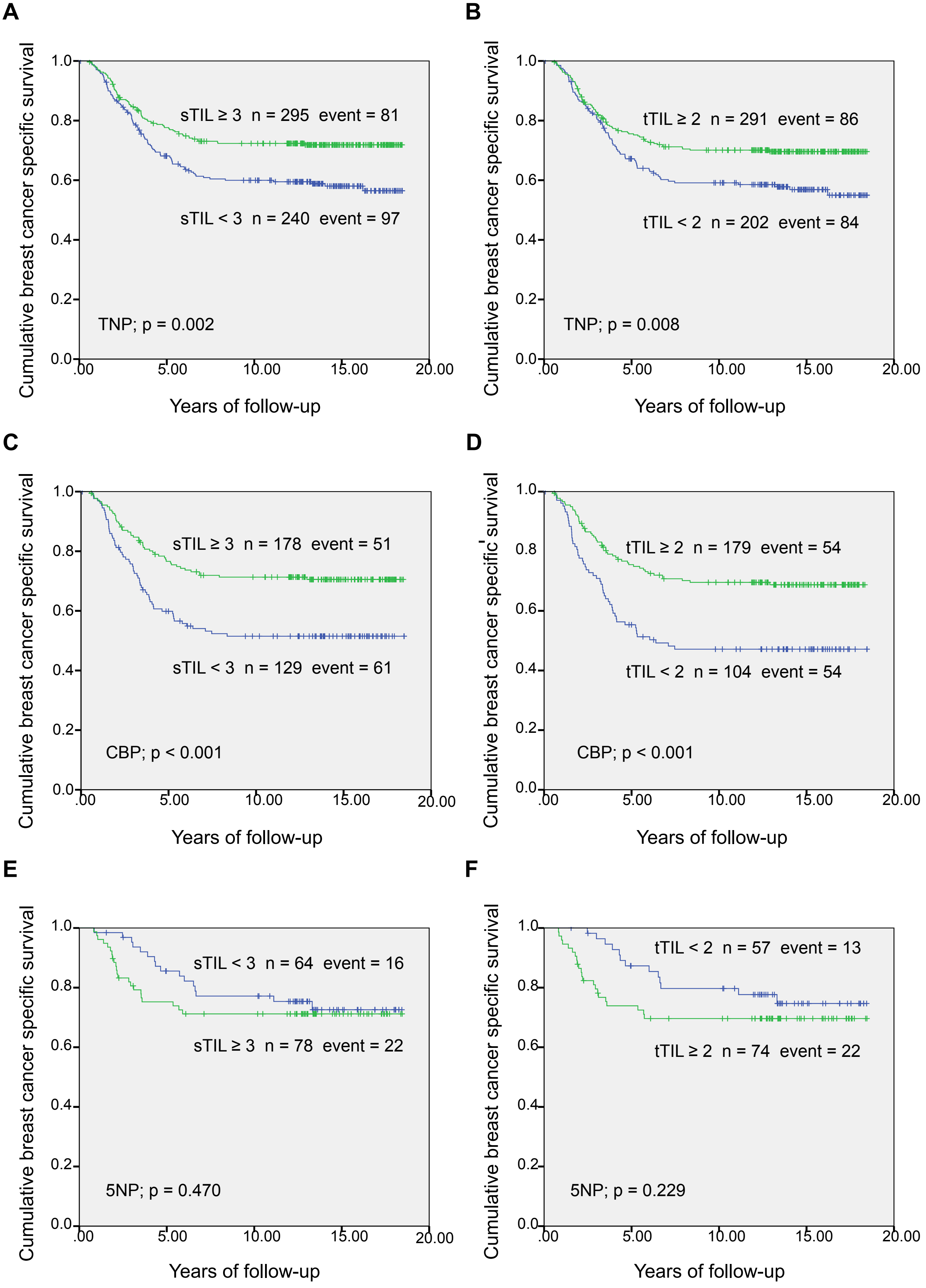

Figure S3. Breast cancer specific survival (BCSS) by sTIL and tTIL in different breast cancer intrinsic subgroups. (A) sTIL in triple negative (TNP), (B) tTIL in TNP, (C) sTIL in core basal (CBP), (D) tTIL in CBP, (E) sTIL in five negative (5NP), and (F) tTIL in 5NP subgroup.
