# Supplementary material for: CD8+ lymphocyte infiltration is an independent favorable prognostic indicator in basal-like breast cancer
Source: Breast Cancer Res. 2012 Mar 15;14(2):R48. doi: 10.1186/bcr3148 (PMC3446382; doi:10.1186/bcr3148)
Supplement: Additional file 6 — Correlation of re-scoring of CD8+ TILs by the same and different pathologists. The scatter plots demonstrated correlations of repeated scoring for 490 cases by the same pathologist for CD8+ iTIL (A) and sTIL (B), and re-scoring of CD8+ iTIL for 200 cases by two pathologists (C). [file bcr3148-S6.PDF]

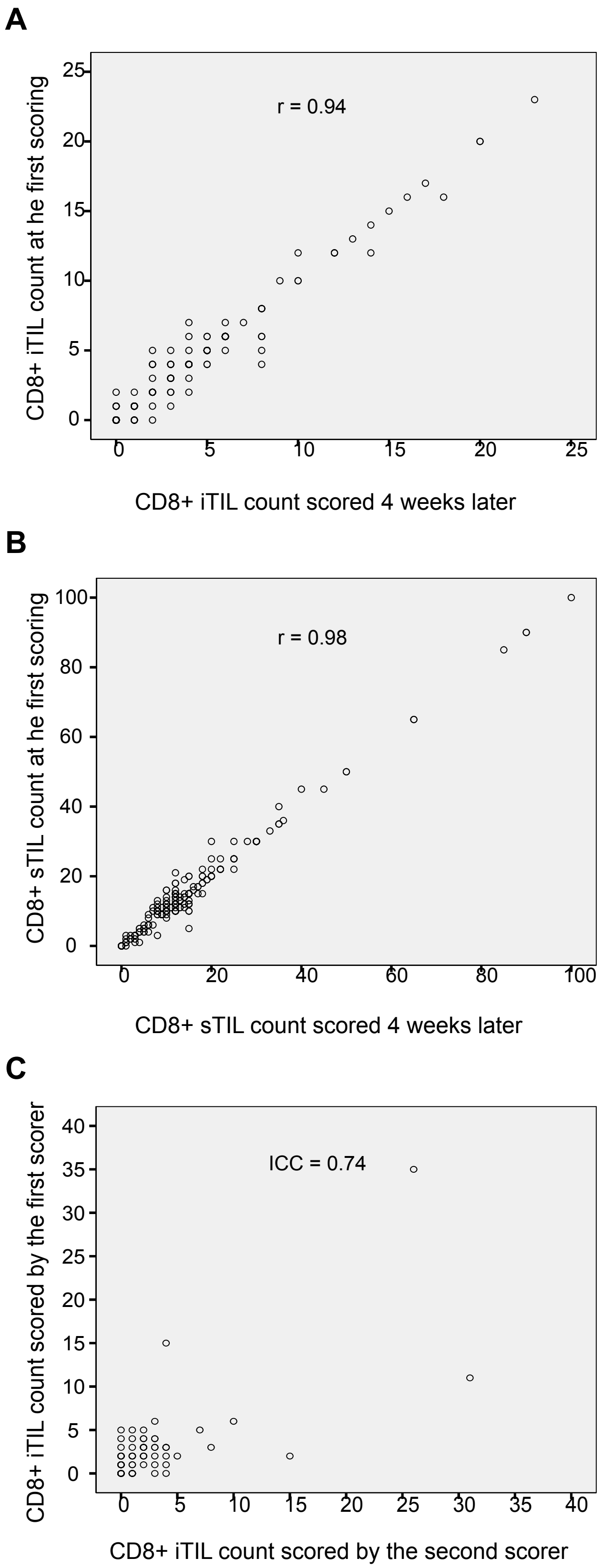

Figure S4. Correlation of re-scoring of CD8+ TILs by the same and different pathologists. Repeated scoring for 490 cases by the same pathologist for CD8+ iTIL (A) and sTIL (B); and re-scoring of CD8+ iTIL for 200 cases by two pathologists (C).
