# Supplementary material for: CD8+ lymphocyte infiltration is an independent favorable prognostic indicator in basal-like breast cancer
Source: Breast Cancer Res. 2012 Mar 15;14(2):R48. doi: 10.1186/bcr3148 (PMC3446382; doi:10.1186/bcr3148)
Supplement: Additional file 7 — Relapse-free survival (RFS) by iTIL among groups with different age and ER status. Kaplan-Meier function survival analysis of association between iTIL and RFS in: (A) age < 50 year, (B) age ≥ 50 year; (C) ER-, and (D) ER+. [file bcr3148-S7.PDF]

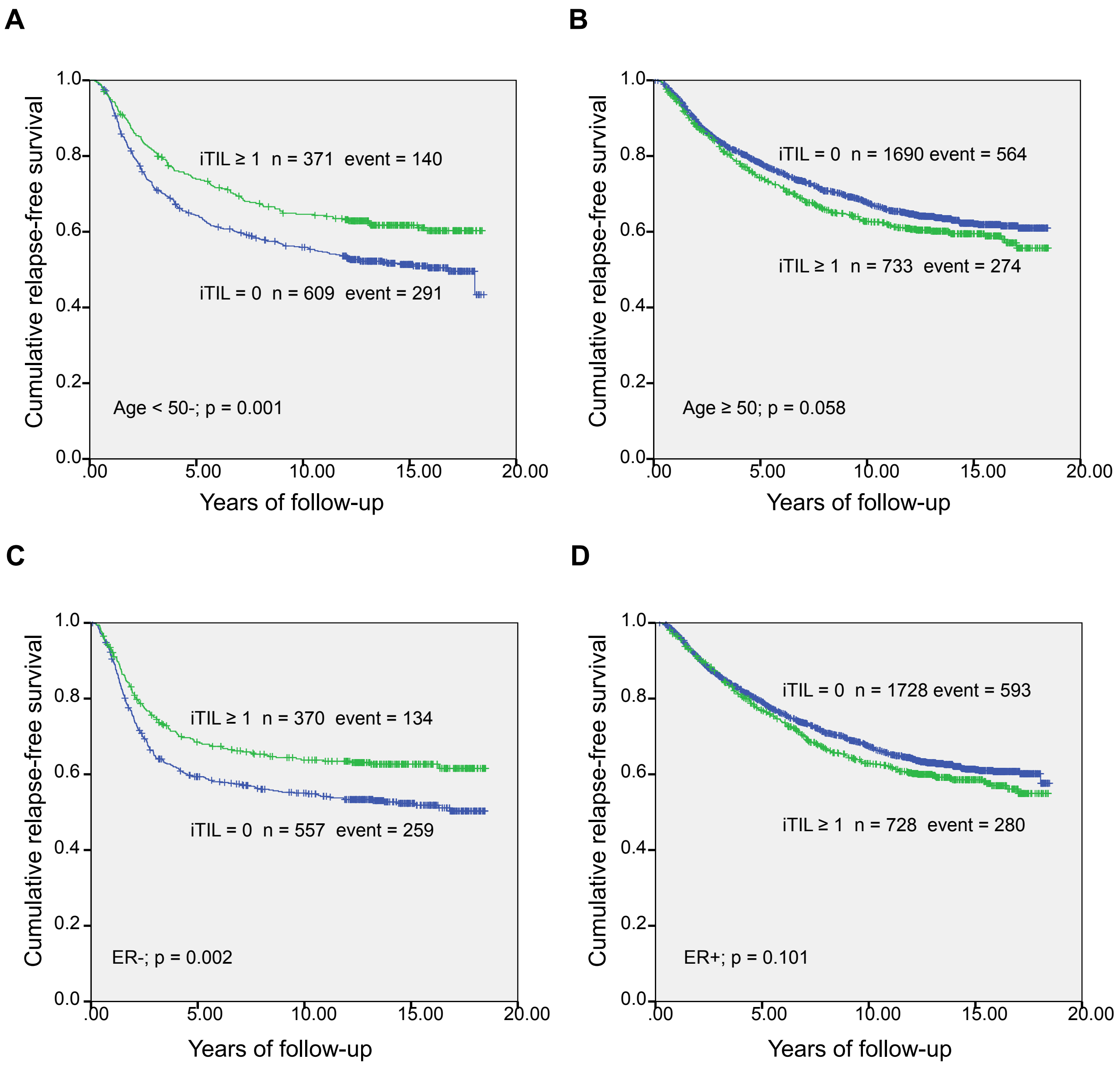

Figure S5. Relapse-free survival (RFS) by iTIL among groups with different age and ER status. (A) age < 50 year, (B) age ≥ 50 year; (C) ER-, and (D) ER+.
