# Supplementary material for: CD8+ lymphocyte infiltration is an independent favorable prognostic indicator in basal-like breast cancer
Source: Breast Cancer Res. 2012 Mar 15;14(2):R48. doi: 10.1186/bcr3148 (PMC3446382; doi:10.1186/bcr3148)
Supplement: Additional file 8 — Relapse-free survival (RFS) by iTIL in different breast cancer intrinsic subgroups. Kaplan-Meier function survival analysis of association between iTIL and RFS in: (A) luminal A, (B) luminal B, (C) HER2+/ER-, (D) Triple negative, (E) core basal, and (F) five negative subgroups. [file bcr3148-S8.PDF]

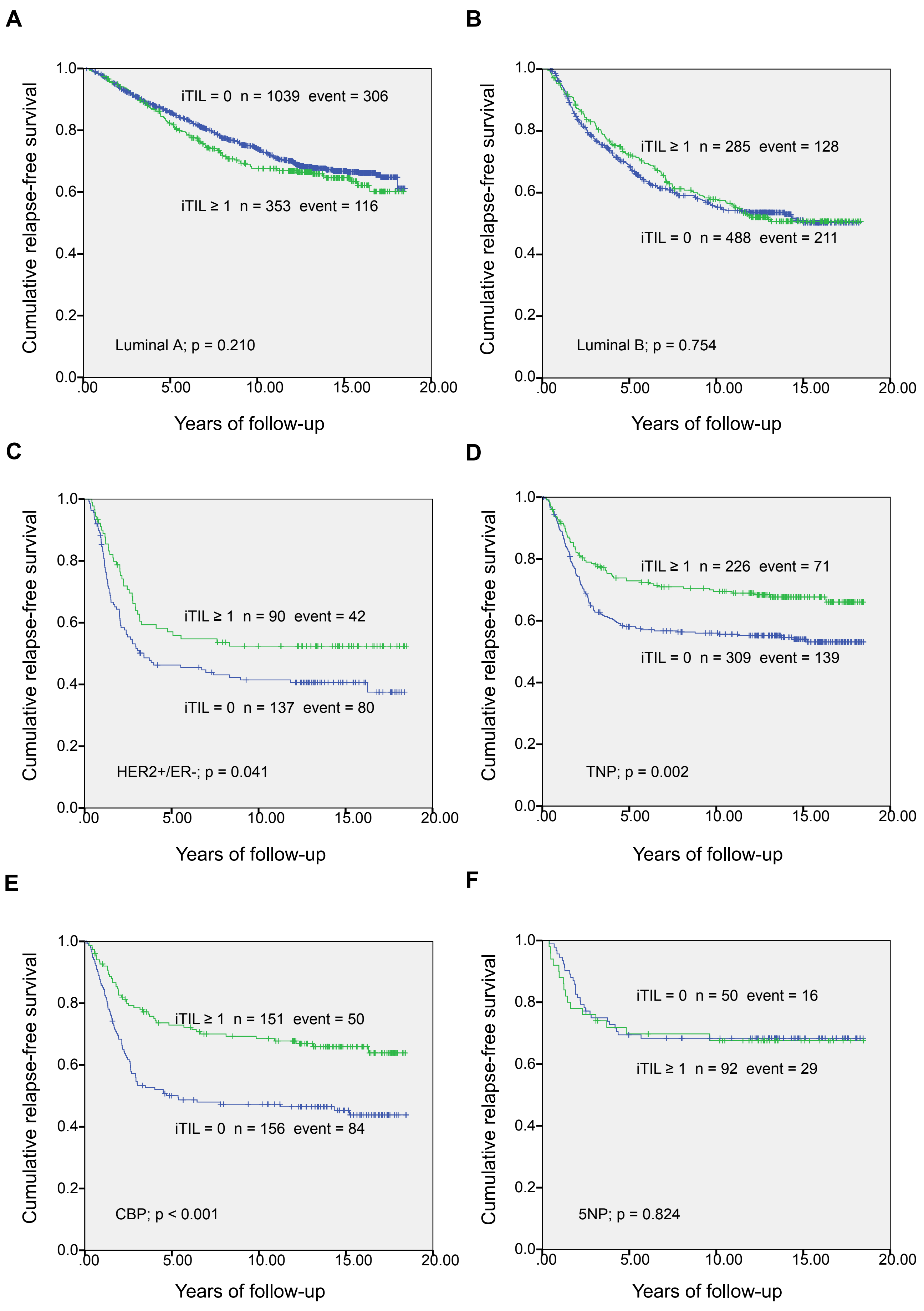

Figure S6. Relapse-free survival (RFS) by iTIL in different breast cancer intrinsic subgroups. (A) luminal A, (B) luminal B, (C) HER2+/ER-, (D) triple negative (TNP), (E) core basal (CBP), and (F) five negative (5NP) subgroups.
